# Tropical basin interactions reduce spring predictability barrier of ENSO in a deep learning model

**DOI:** 10.1126/sciadv.aeb0901

**Published:** 2026-05-20

**Authors:** Lu Zhou, Rong-Hua Zhang

**Affiliations:** ^1^ State Key Laboratory of Climate System Prediction and Risk Management/Key Laboratory of Meteorological Disaster, Ministry of Education/Collaborative Innovation Center on Forecast and Evaluation of Meteorological Disasters/School of Marine Sciences, Nanjing University of Information Science and Technology, Nanjing 210044, China.; ^2^ Laoshan Laboratory, Qingdao 266237, China.

## Abstract

The El Niño–Southern Oscillation (ENSO) exhibits a pronounced decline in predictability during boreal spring, referred to as spring predictability barrier (SPB). While tropical basin interactions among the Indian, Atlantic, and Pacific Oceans potentially enhance ENSO predictability, their roles in mitigating SPB within deep learning (DL) frameworks remain underutilized. Here, we introduce GL-Geoformer, a DL model for global tropical ocean-atmosphere prediction. GL-Geoformer captures spatiotemporal evolutions of wind and three-dimensional temperature anomalies across the tropical basins. Our modeling demonstrates that incorporating tropical basin interactions substantially reduces SPB, enabling GL-Geoformer to achieve skillful ENSO predictions up to 16 months in advance when initiated in spring. Pacemaker experiments are performed to quantify individual and synergistic contributing nonlinearities of Indian Ocean Dipole and Atlantic Niño via subsurface heat transport and Walker circulation mechanisms, respectively. This study provides a data-driven framework to represent tropical basin interactions and reduce SPB, thereby deepening understanding of ENSO predictability.

## Teaser

Deep learning captures interbasin interactions and demonstrates their role in reducing the ENSO spring predictability barrier.

## INTRODUCTION

El Niño–Southern Oscillation (ENSO) is the most dominant interannual climate mode, produced by basin-scale ocean-atmosphere interactions in the tropical Pacific. It manifests as irregular fluctuations between warm (El Niño) and cold (La Niña) sea surface temperature (SST) conditions in the equatorial Pacific (*1*), with far-reaching global consequences for environmental and socioeconomic systems through atmospheric teleconnections and oceanic pathways (*2*–*6*). Despite substantial progress in understanding ENSO dynamics and improving predictions over past decades (*7*–*9*), prediction skill remains limited beyond 1 year. A central challenge is the spring predictability barrier (SPB), where prediction accuracy sharply declines for predictions initialized from February to May (*10*–*12*).

Growing evidence underscores that ENSO is embedded within a tightly coupled tropical climate system, where interactions among the Pacific, Indian, and Atlantic Oceans—termed tropical basin interactions—profoundly modulate its evolution and predictability (*13*–*17*). Capturing these interactions is thus critical for overcoming the SPB and effectively improving ENSO prediction skills. Recently, Zhao *et al.* (*18*) introduced an extended nonlinear recharge oscillator (XRO) model, a physics-based framework that rigorously quantifies how interactions across tropical basins and among major climate modes enhance ENSO predictability and mitigate the SPB. While being not an AI-based approach, this XRO, with the underlying mechanisms being explicitly and manually represented and tuned, achieves prediction skills comparable to state-of-the-art deep learning (DL) models (*19*, *20*), which provides a crucial benchmark and a physically explainable account of interbasin processes associated with ENSO modulations.

However, the relative and synergistic contributions of individual basins to ENSO predictability remain difficult to fully quantify, partly because of observational limitations and the inherent challenges of isolating these complex, nonlinear interactions. Traditional prediction frameworks, including both dynamical and statistical models, often struggle to adequately represent these interbasin influences because of issues like initialization drift, model bias, or insufficient model complexity (*21*, *22*).

Data-driven DL techniques offer a promising alternative by learning dynamical relationships directly from observational or simulated data. DL models have demonstrated superior performance in long-lead ENSO predictions (*19*, *20*, *23*). However, previous DL studies have predominantly focused on Pacific SST indices or regional tropical Pacific data, largely neglecting the explicit role of tropical basin interactions. It therefore remains an open question whether DL models can intrinsically learn these complex interbasin dynamics and, if so, how such learned interactions contribute to overcoming the SPB within a data-driven paradigm.

Here, we develop a transformer-based DL model for the global tropical ocean-atmosphere system, termed GL-Geoformer (see Materials and Methods and texts S1 and S2), which simultaneously predicts monthly sea surface wind and three-dimensional (3D) ocean temperature fields across the global tropics (0°–360°, 32°S–32°N). Trained on historical simulations from CMIP6 (*24*), the model uses 9-month sequences of surface wind stress and 3D ocean temperature anomalies as input predictors (totally 13 variables) to generate 24-month multivariate predictions for the same 13 variables. Its recursive rolling prediction strategy preserves temporal continuity and physical consistency in ocean-atmosphere coupling, which is crucial for capturing key ENSO dynamics (e.g., Bjerknes feedback). Through controlled ablation and pacemaker experiments, we systematically quantify how interactions among the tropical Pacific, Indian, and Atlantic Oceans individually and synergistically reduce the SPB and enhance ENSO prediction skill, particularly for predictions initiated in early boreal year. For instance, the model produces accurate hindcasts of the 2020 to 2021 strong La Niña nearly 1 year in advance and elucidates distinct remote mechanisms from each basin responsible for the evolution of SST anomalies in the tropical Pacific.

In concert with the physics-based and fine-tuned XRO framework (*18*), our data-driven GL-Geoformer also demonstrates that representing interbasin processes is key to advancing ENSO predictability. Together, these complementary approaches, i.e., one rooted in simplified physical modeling and the other in high-dimensional DL, offer a viable alternative pathway to improve long-range predictions and deepen the physical understanding of ENSO modulations resulting from tropical basin interactions and its predictability barriers.

## RESULTS

### Multivariate ENSO prediction skills

To evaluate the predictive performance of GL-Geoformer, we apply it to an independent test set using Global Ocean Data Assimilation System [GODAS; (*25*, *26*)], whose reanalysis data span from 1980 to 2023. The model predicts 13 variables, including zonal and meridional wind stress components (τ_x_ and τ_y_) and ocean temperature anomalies across 11 levels. To quantify and reduce single-model uncertainty inherent to neural network training, we construct an ensemble comprising 10 model realizations with identical architectural specifications but distinct random initialization seeds. All experimental results and performance metrics are represented as ensemble averages across these realizations.

**Fig. 1.**
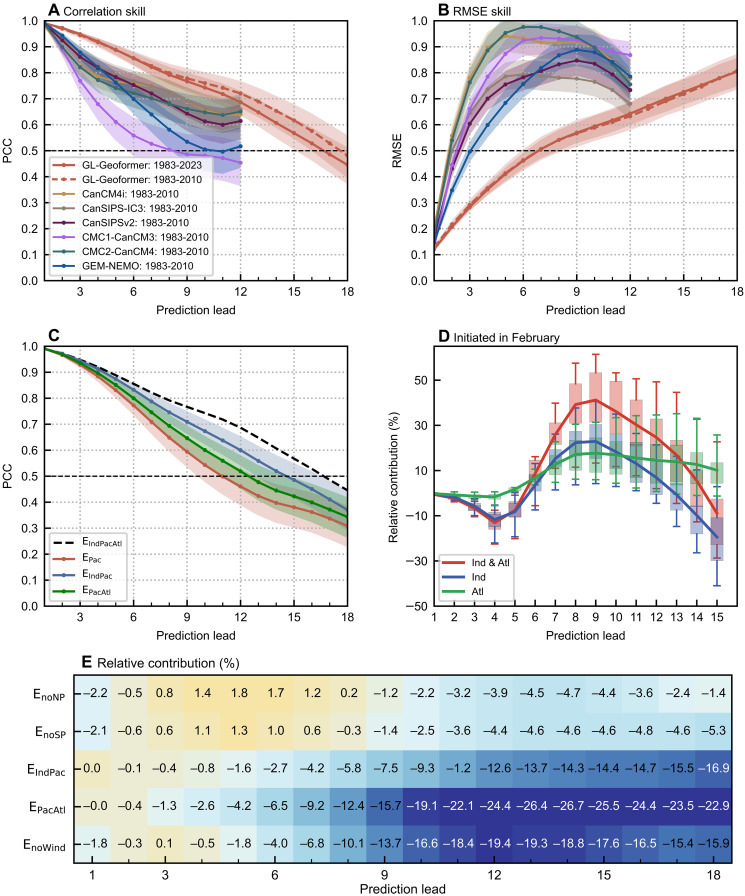
Prediction skill assessment of ENSO and analysis of ocean basin contributions to its predictability. (**A** and **B**) All-months PCC and RMSE skills for 3-month running mean Niño3.4 indices as a function of prediction lead months. Solid and dashed red lines represent GL-Geoformer skill from 1983 to 2010 and 1983 to 2023, respectively; other colored lines represent six climate models from NMME products from 1983 to 2010. (**C**) All-months PCC skill for the Niño3.4 index in the control experiment (E_IndPacAtl_, black dashed line) and three sensitivity experiments from 1983 to 2023: E_Pac_ (red line; the effects from both Indian and Atlantic Oceans are removed), E_IndPac_ (blue line; Atlantic Ocean effects removed), and E_PacAtl_ (green line; Indian Ocean effects removed). The shading around the lines in (A) to (C) denotes the 95% confidence level. (**D**) Relative contribution of Indian Ocean (blue), Atlantic Ocean (green), and their synergy effects (red) to Niño3.4 correlation skill for February initialization predictions. Box plots are based on 10 ensemble members; solid lines indicate the ensemble mean. (**E**) Impact of the removal of basin-specific forcings on Niño3.4 PCC prediction skill relative to the control experiment. Shading represents the relative percentage change, and the numeric values in each block denote the corresponding percentage change. Positive (negative) values indicate enhanced (reduced) prediction skill in the sensitivity experiments. The experiments are defined as follows: E_noWind_, prediction with the influence of surface wind fields removed; E_noSP_ and E_noNP_, predictions with the influences of the South Pacific (120°E–80°W, 32°S–10°S) and the North Pacific (120°E–80°W, 10°N–32°N) removed, respectively.

The GL-Geoformer effectively predicts Niño3.4 SST anomalies with lead times exceeding 17 months when using the Pearson correlation coefficients (PCC) of 0.5 as a criterion (Fig. 1A). During the test period from 1983 to 2010, the all-season correlation skill of the Niño3.4 index is much higher than the dynamical prediction models from North American Multi-Model Ensemble [NMME; (*27*)] products. Moreover, the ENSO intensity predicted by the GL-Geoformer, as quantified by root mean square error (RMSE) skills, is more consistent with observations and outperforms dynamical models at any lead time within 1 year (Fig. 1B). Beyond Niño index predictions, we also evaluate its predictive capability for global tropical multivariate fields; comprehensive evaluations are provided in figs. S1 to S6.

To further quantify the relative contributions of different ocean basins to ENSO predictability, a series of ablation experiments are conducted using the GL-Geoformer. In the control experiment in which effects from all the Indian (Ind), Pacific (Pac), and Atlantic (Atl) Oceans are included (denoted as E_IndPacAtl_), predictions are made using the model for all variables from global tropics as input predictors. Then, we sequentially or simultaneously eliminate the effects of anomaly fields in the Indian Ocean (30°E–120°E, 32°S–32°N)/Atlantic Ocean (80°W–30°E, 32°S–32°N) from both the input predictors and the rolling prediction procedure; these experiments are denoted as E_PacAtl_ (Indian Ocean effects are excluded), E_IndPac_ (Atlantic Ocean effects are excluded), and E_Pac_ (both basin effects are excluded), respectively. As shown in Fig. 1C for ENSO prediction, the exclusion of Indian Ocean effects reduces the effective lead time from 17 months (in E_IndPacAtl_) to 12 months, whereas that of Atlantic Ocean effects reduces it to 15 months. Simultaneous exclusion of both basins results in a further decline in prediction skill to 11 months. These results quantitatively demonstrate that cross-basin remote effects from both the Indian and Atlantic Oceans individually make contributions to enhanced ENSO predictability within this DL modeling framework, with the Indian Ocean exerting a substantially larger influence (also see figs. S6 and S7).

We further quantify the relative contributions of individual variables to seasonal ENSO prediction skill. As illustrated in Fig. 1E, sea surface wind stress contributes more than 10% to ENSO prediction skill for lead times exceeding 8 months, with its contributions increasing to ~20% at 12-month leads. Variables from the extratropical Pacific demonstrate limited predictive utility, contributing less than 10% to skill at longer lead times. In addition, both the effects from Indian and Atlantic Oceans enhance ENSO predictability at lead times exceeding 6 months, with their maximum contributions seen at approximately 12- to 15-month leads. Specifically, at a 14-month lead time, the Indian Ocean accounts for ~27% of PCC prediction skill, while the Atlantic Ocean contributes ~14%. Furthermore, we extend this analysis to evaluate the individual versus synergistic contributions of the Indian and Atlantic Oceans (table S1 and fig. S7). Our results clearly demonstrate that the predictive gain arising from combined interbasin interactions exceeds the simple linear superposition of their individual effects analysis during the entire period from 1983 to 2023. This nonlinearity highlights that synergistic coupling among the three basins plays a critical role in long-term ENSO predictability, consistent with the interbasin interaction mechanisms recently proposed by Zhao *et al.* (*18*) and Fan *et al.* (*16*).

### Attributions of basin interactions to reducing the SPB

**Fig. 2.**
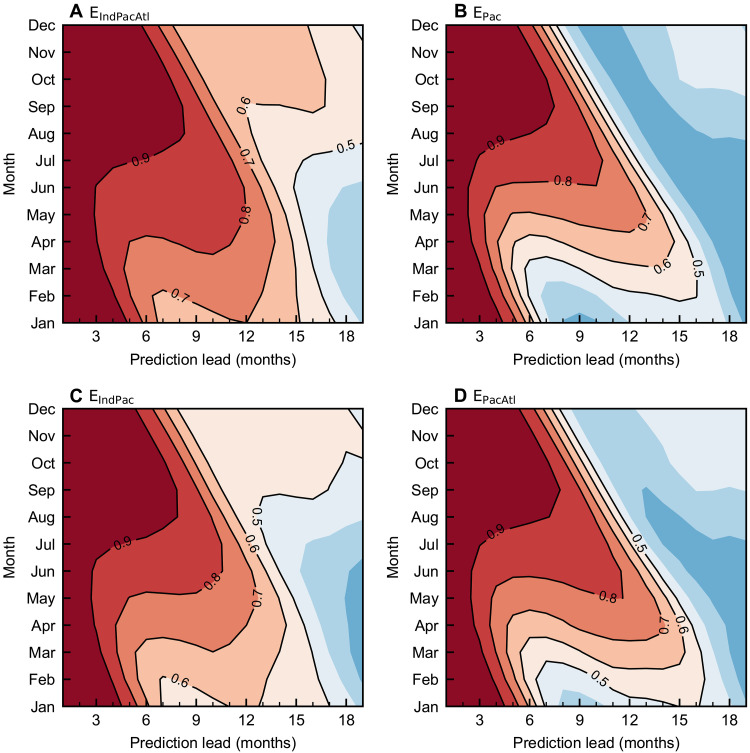
ENSO correlation skill in different sensitivity experiments. Niño3.4 SST anomaly correlation skill of predictions initiated in each calendar month from 1983 to 2023 for (**A**) E_IndPacAtl_, (**B**) E_Pac_, (**C**) E_IndPac_, and (**D**) E_PacAtl_ experiments. Contours indicate skill values exceeding 0.5.

The GL-Geoformer shows a notable enhancement in spring-time ENSO prediction skills, extending beyond 16 months for effective predictions when initiated in February to May (Fig. 2A), which is a period recognized as the most challenging season due to the SPB. This substantial improvement as compared to dynamical models (fig. S8) is attributed to interbasin influences, which can be tested using this model. For example, when the GL-Geoformer includes only the Pacific Ocean (Fig. 2B), the model exhibits substantially reduced ENSO prediction skill across all seasons, with the effective lead time diminishing to ~6 months in springtime-initialized predictions. The incorporations of Indian and Atlantic Oceans into the model lead to varying degrees of improvement in ENSO prediction skills (Fig. 2, C and D). The inclusion of the Indian Ocean dramatically enhances spring ENSO prediction capability, extending the effective lead time to approximately 15 months (Fig. 2C). While the individual contribution of the Atlantic Ocean is relatively modest, its synergistic interaction with the Indian Ocean helps amplify the overall improvement in prediction skill (Fig. 2A).

To quantify the individual and synergistic impacts of the Indian and Atlantic Oceans on mitigating SPB, we compare the Niño3.4 prediction correlation skills from the E_IndPac_, E_PacAtl_, and E_IndPacAtl_ experiments with those from the E_Pac_ experiment. As shown in Fig. 1D, at short-term predictions made from February initial conditions, both the Indian Ocean alone and the combined Indian-Atlantic interactions exhibit negative contributions, reaching as low as −10 to −15%; this indicates that including these remote effects initially degrades prediction skill. The counterintuitive phenomenon suggests that cross-basin signals may introduce noise information during the short-term prediction period, highlighting that ENSO predictability at short lead times primarily originates from local processes within the tropical Pacific. However, a reversal occurs as lead times exceed 6 months, coinciding with the typical onset of the SPB. The combined tropical basin interactions (red line in Fig. 1D) demonstrate substantial improvements, with contributions exceeding 40% at lead times of 8 to 10 months, which precisely corresponds to the challenging boreal spring-summer period when the SPB typically manifests. The Indian Ocean effect alone follows a similar pattern but with more moderate enhancements of 25 to 30%. Notably, the Atlantic Ocean effect maintains relatively stable positive contributions of 10 to 20% across most lead times, showing less sensitivity to the SPB timing. These findings indicate that basin remote influences are particularly valuable for overcoming the SPB, with their beneficial effects most pronounced during the critical 6- to 12-month lead time prediction, when traditional approaches struggle most.

### Process understanding and interpretability analyses

Although the ablation experiments and skill assessments establish the statistical importance of extra-Pacific basins in ENSO prediction, demonstrating physical causality requires additional mechanistic evidence beyond correlation-based analysis. Notably, we conduct three experiments within the GL-Geoformer that emulate the forced-response and pacemaker experiments commonly used in dynamical modeling.

First, to evaluate whether the data-driven model faithfully represents the ocean’s response to atmospheric forcing, we perform an ~40-year ocean-only integration (1980 to 2023) driven solely by observed wind stress. This assesses the model’s capability in representing the ocean’s dynamical response and its internal ocean-atmosphere coupling mechanisms. Second, we perform two pacemaker experiments in which SST anomalies in the tropical Indian (30°E–120°E 32°S–32°N) and Atlantic Oceans (80°W–30°E, 32°S–32°N) are respectively restored to observational values and then integrate the GL-Geoformer from 1980 to 2023. Crucially, to isolate the causal impact of a specific basin, the multivariate variability in the other non-Pacific basin (e.g., masking the Atlantic variables while restoring the Indian Ocean SST anomalies) is intentionally suppressed by setting all its variables to zero. To minimize the influence of the model initial conditions, all subsequent analyses are based on the final 30 years (1994 to 2023) of the integration period.

**Fig. 3.**
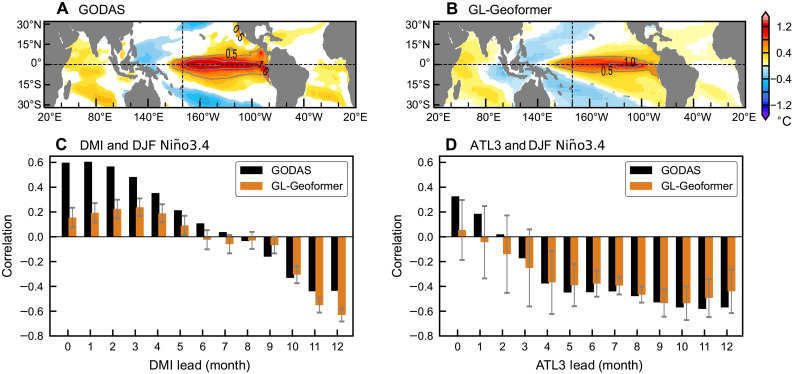
Physical consistency and cross-basin response analysis of the GL-Geoformer. (**A** and **B**) Regression of the DJF Niño3.4 index onto the SST anomaly fields in GODAS reanalysis and GL-Geoformer free-running simulations forced by observed wind stress; only regions exceeding 95% confidence level are shown. (**C** and **D**) Lead correlation between DMI and DJF Niño3.4 index, and ATL3 and DJF Niño3.4 index, from GODAS reanalysis (black) and pacemaker experiment with Indian Ocean [(C), orange] or Atlantic Ocean [(D), orange] SST anomalies nudged to observations. Error bars indicate the 95% confidence interval of the multimodel mean, estimated from intermodel variability using a Student’s *t* distribution. Simulations span 1980 to 2023 with analysis focused on the final 30 years (1994 to 2023).

In the atmospheric forcing-constrained experiment, the regression pattern of the simulated December-January-February (DJF) Niño3.4 index onto global SST anomalies reveals a classical ENSO-driven teleconnection pattern (Fig. 3B), which is consistent with observational results (Fig. 3A). The observational analysis indicates that the largest positive regression coefficients are concentrated in the central-eastern equatorial Pacific, accompanied with negative values in the western Pacific and off-equatorial subtropical Pacific regions (Fig. 3A). The GL-Geoformer simulation successfully captures this ENSO-like dipole structure in the tropical Pacific and the associated zonal SST anomaly gradients (Fig. 3B), suggesting realistic representation of tropical Pacific ocean-atmosphere coupling processes. Furthermore, the positive SST anomaly regression coefficients over the tropical Indian and Atlantic Oceans both in simulations and observations further reflect ENSO global teleconnections. The excessively broad region of weaker negative coefficients in the tropical Indo-Pacific (Fig. 3B) may be attributed to biases in simulating the Indonesian Throughflow strength or the Pacific cold tongue representation, which are known challenges in coupled climate models (*28*, *29*).

The Indian Ocean SST anomaly pacemaker experiment demonstrates that the GL-Geoformer effectively captures the fundamental teleconnection between the two basins (Fig. 3C). Specifically, the model successfully reproduces the evolving trend of the lead-lag correlation between the Dipole Mode Index (DMI; the SST anomaly difference between the western (50°E–70°E, 10°S–10°N) and southeastern (90°E–110°E, 10°S–0°) tropical Indian Ocean) and the DJF Niño 3.4 index (Fig. 3C), confirming its ability to internalize trans-basin signals. The differences in correlation intensity at short versus long lead times reflect the respective contributions of the fast atmospheric response and slow oceanic heat content adjustment to ENSO evolution under Indian Ocean SST anomaly forcing. Specifically, Indian Ocean SST anomaly can rapidly trigger western Pacific wind anomalies through modifications of the Walker circulation. This atmospheric bridge mechanism operates instantaneously but induces relatively weak SST responses in the central-eastern equatorial Pacific, thus manifesting as weaker correlations at shorter leads in our experiments. In contrast, strong coupling and sustained influences typically involve the propagation of oceanic waves and the redistribution of subsurface heat content. These processes require longer adjustment timescales and result in higher correlations between DMI and SST anomaly in Niño3.4 region at long leads (Fig. 3C).

In contrast, because of the absence of direct oceanic connectivity between the tropical Atlantic and Pacific Oceans, the influence from the tropical Atlantic on ENSO is primarily realized via atmospheric teleconnections. Therefore, the degree of its effects on ENSO remains relatively insensitive to inconsistencies between restored SST and freely evolving subsurface temperatures in the pacemaker experiments. As shown in Fig. 3D, the GL-Geoformer accurately reproduces the observed negative correlation between the spring-summer ATL3 SST anomalies (20°W–0°, 3°S–3°N) and subsequent winter Niño3.4 variability. These successful depictions demonstrate not only the model ability to realistically capture tropical ocean-atmosphere interactions but also its superior performance compared to previous DL-based modeling, which is typically limited to single-variable or regional-scale simulations.

### A case study for the 2020 to 2021 strong La Niña event

In addition to these overall assessments, a representative case study is conducted to clearly illustrate how interbasin processes influence ENSO prediction during spring. A particularly illustrative example is taken for the prolonged cold phase in the tropical Pacific from 2020 to 2022, which featured a rare triple-dip La Niña during the winters of 2020, 2021, and 2022, respectively. Among these, the 2020 La Niña event stands out as the fifth strongest one since the 1990s (the other four being 1998 to 1999, 1999 to 2000, 2007 to 2008, and 2010 to 2011 La Niña); it is unique in its developing evolution from near-neutral initial conditions (fig. S9). This event contradicts the classical ENSO paradigm, which typically associates multiyear La Niña events with preceding strong El Niño (*30*). That is, the 2019 El Niño is relatively weak, and the equatorial Pacific warm water volume in 2020 is the weakest among these five events (*31*), thereby failing to provide a sufficiently large oceanic heat discharge typically associated with subsequent prolonged La Niña conditions (*32*, *33*).

Most climate models greatly underestimated the intensity in predicting this event based on initial conditions from spring 2020 (*10*, *34*). For instance, the ensemble mean predictions of Niño3.4 SST anomalies for October to December 2020, based on predictions initialized in mid-April 2020, range between −0.2° and −0.6°C (fig. S10), whereas the observed anomaly is approximately −1.3°C. This predictive bias raises two questions: What processes drive the unusually strong 2020 to 2021 La Niña event? Do the tropical Indian and Atlantic Oceans contribute substantially to its development?

To quantify the role of tropical basin interactions in the development and predictability of the 2020 to 2021 La Niña, we conduct a set of sensitivity experiments following the methodology outlined above, namely E_IndPacAtl_, E_IndPac_, E_PacAtl_, and E_Pac_. The model uses multivariate anomalies from July 2019 to March 2020 (a 9-month time interval) as initial conditions with a rolling integration strategy to predict the evolution of global tropical multivariate in the following 24 months.

**Fig. 4.**
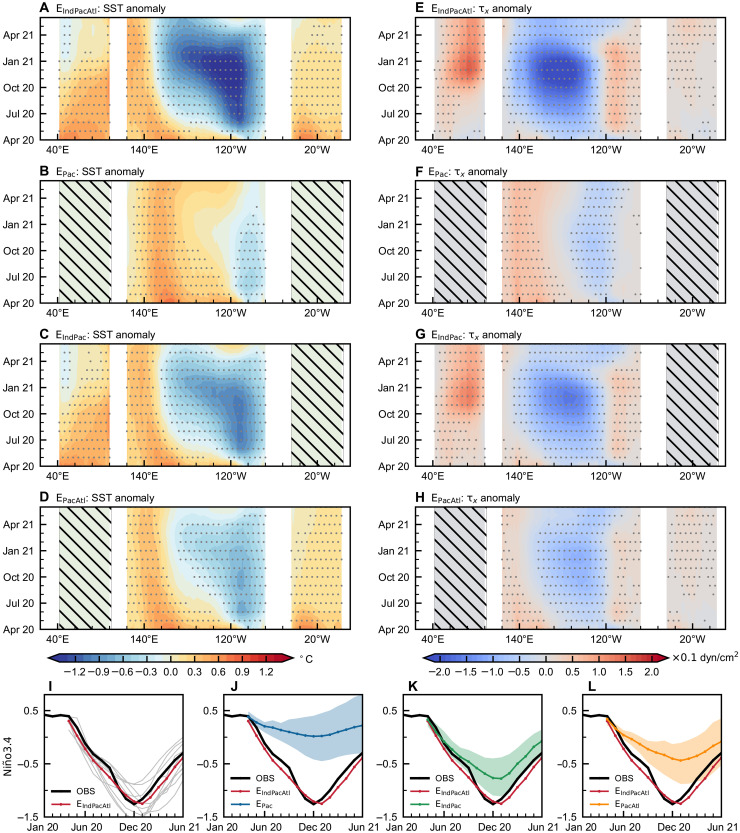
Interbasin effects on the evolution of the 2020 to 2021 La Niña event. (**A** to **D**) Zonal-time sections along the equator for SST anomalies (shading, with stippling indicating significance at the 95% confidence level) predicted using the GL-Geoformer from initial conditions in April 2020 in E_IndPacAtl_, E_Pac_, E_IndPac_, and E_PacAtl_, respectively. (**E** to **H**) The same as (A) to (D) but for zonal wind stress anomalies (τ_x_). The black hatched regions in (A) to (H) indicate areas where the effects of tropical Indian and Atlantic Oceans are removed from the predictions. (**I** to **L**) Reanalyzed (black line) Niño3.4 SST anomalies and predicted (colored lines) ones using the GL-Geoformer initiated in April 2020. Light-gray lines in (I) denote the predictions from 10 ensemble members, and thick red lines in (I) to (L) represent the ensemble mean in E_IndPacAtl_. The blue line in (J), green line in (K), and orange line in (L) represent ensemble predictions in E_Pac_, E_IndPac_, and E_PacAtl_, respectively; shading represents the 95% confidence level.

**Fig. 5.**
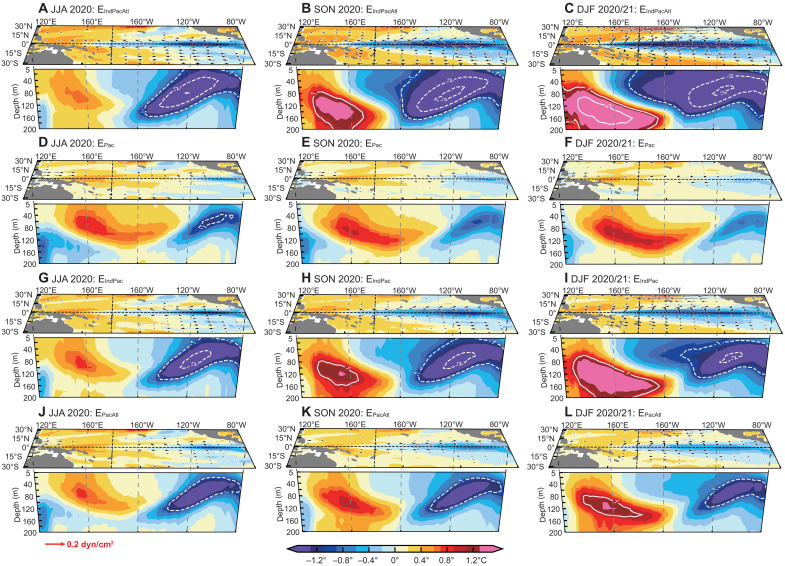
Tropical Pacific predictions for the 2020 to 2021 La Niña event from different ocean basin configurations. Predictions are made for June 2020 to February 2021 from initial conditions in April 2020 using the GL-Geoformer. Four experimental results from (**A** to **C**) E_IndPacAtl_, (**D** to **F**) E_Pac_, (**G** to **I**) E_IndPac_, and (**J** to **L**) E_PacAtl_ are presented: The top panel shows surface wind stress (vectors) and SST anomalies (shading) in the horizontal sections; the bottom panel shows upper-ocean temperature anomalies at the equator in the vertical-zonal sections (shading and contours).

As demonstrated in Fig. 4A, the control experiment (E_IndPacAtl_) accurately predicts the strong La Niña event that occurs in winter 2020 when initiated in April 2020, with the ensemble mean intensity of Niño3.4 SST anomaly closely matching to observations (Fig. 4I). The GL-Geoformer accurately reproduces key atmospheric and oceanic anomalies, including persistent easterly wind anomalies over the central-western equatorial Pacific and westerly wind anomalies over the equatorial Indian Ocean from 2020 to 2021, as well as the sustained warming of the tropical Indian Ocean in 2020 (Figs. 4, A and E, and 5, A to C). Notably, most physics-driven climate models initialized in spring of 2020 systematically underestimate the intensity of this event, with predicted Niño3.4 SST anomaly amplitudes reaching only about one-third of the observations (fig. S10).

When both the Indian and Atlantic Ocean influences are removed from the model input fields, the GL-Geoformer largely fails to predict the 2020 to 2021 La Niña event (Figs. 4, B and J, and 5, D to F). In contrast, E_IndPac_ demonstrates markedly improved predictive skill relative to E_Pac_ when Indian-Pacific interactions are incorporated in GL-Geoformer predictors (Figs. 4, C and K, and 5, G to I). In this configuration, the predicted Niño3.4 SST anomaly reaches −0.75°C in winter 2020, achieving nearly 60% of the intensity predicted by the control experiment but still showing substantially reduced prediction uncertainty (Fig. 4K). Compared to the tropical Indian Ocean, the tropical Atlantic contributes less to the 2020 to 2021 La Niña development (table S2). Incorporating Atlantic multivariate fields into the GL-Geoformer yields only modest prediction improvements over E_Pac_, with strengthened easterlies over the central-eastern Pacific and Niño3.4 SST anomalies reaching −0.4°C (∼30% of the control experiment intensity; Figs. 4, D, H, and L, and 5, J to L). A detailed analysis of the effects of individual basin processes on the 2020 to 2021 La Niña event is provided in text S3 and figs. S11 to S13.

## DISCUSSION

Recent advances in DL models offer improved performance over traditional methods for ENSO prediction, yet the persistent SPB remains a fundamental challenge limiting seasonal predicting capabilities. This study presents GL-Geoformer, a transformer-based DL model that designed to overcome the SPB through explicit representation of global tropical ocean-atmosphere interactions. GL-Geoformer is a DL model that is built to integrate wind stress and 3D ocean temperature anomalies in the global tropics, capturing both individual and synergistic influences from the Indian and Atlantic Oceans that prove crucial for spring ENSO predictability. Our analyses reveal that Pacific-only configurations are limited by the traditional SPB, as springtime-initialized predictions degrade rapidly beyond 6 months. In contrast, incorporating tropical basin interactions markedly extends the prediction skill, allowing for accurate forecasts up to 16 months in advance.

Sensitivity experiments reveal processes responsible for the temporal evolution of SPB mitigation: Interbasin contributions are initially negative at short leads but become increasingly positive beyond 6-month leads, precisely corresponding to a time when the SPB typically manifests. This timing suggests that cross-basin teleconnections provide the sustained predictive information needed to bridge the spring prediction barrier. The success using this purely data-driven approach in reducing the SPB offers valuable insights into how global climate teleconnections can be leveraged to overcome fundamental predictability barriers.

In examining the 2020 to 2021 La Niña, we further show that Pacific-only initialization from spring 2020 fails to predict the event amplitude. However, including Indian and Atlantic signals enables successful hindcasting, with quantitative attribution showing that the Indian Ocean contributes ~60% of event amplitude via subsurface heat transport, while the Atlantic provides ~30% through the Walker circulation modulation. This study also indicates the necessity of taking into account the tropical climate system holistically in ENSO predictions and demonstrates the power of DL models in revealing nonlinear tropical basin interactions that modulate ENSO evolution.

Despite its success, the GL-Geoformer currently uses only surface wind data and is trained exclusively on CMIP6 simulations. Including multilayer atmospheric variables and integrating observational data via transfer learning in future work may further enhance its realism and generalizability.

## MATERIALS AND METHODS

### Datasets

Considering the fundamental dynamical processes of tropical ocean-atmosphere system and especially to ensure the physical completeness of ENSO processes, we specifically select monthly fields of sea surface wind stress (τ_x_ and τ_y_ components) and 11-layer ocean temperature in the upper 200 m (at depths of 5, 20, 40, 60, 80, 100, 120, 140, 160, 180, and 200 m) over the global tropics (0°–360°, 32°S–32°N) as the DL-based training datasets. The training and validation datasets include historical simulations from 30 climate models that participated in the CMIP6 during the period of 1850 to 2014 and reanalysis dataset from Simple Ocean Data Assimilation (SODA) products from 1871 to 1979, and the test datasets consist of GODAS reanalysis during the period of 1980 to 2023 (table S3).

All data inputs into the GL-Geoformer are subject to preprocessing to ensure consistency. First, climatological seasonal cycle and long-term linear trends are removed to eliminate the effects of global warming. Then, the resultant interannual anomalies are interpolated onto regular grids with a resolution of 2° in zonal direction and 0.5° (1°) in meridional direction within (out of) 5°S–5°N. To mitigate the impact of anomalous values during model training, all land areas and missing data points are assigned a value of zero. Considering the magnitude differences between wind stress and ocean temperature anomalies, all data are standardized using the global mean SD before being used for the training and predicting.

During the model pretraining procedure, we select 29 CMIP6 model simulations for training and 1 additional model simulation for validation. Each epoch randomly generates ~10,000 training samples and 250 validation samples, with each sample consisting of input predictors for nine consecutive months. In the subsequent transfer training stage, the GL-Geoformer is trained on simulations from all 30 CMIP6 models, and SODA reanalysis before 1980 are used for validation. Last, the GL-Geoformer is tested using GODAS reanalysis spanning the period from 1980 to 2023, with the model initialized each month to generate predictions for the subsequent 24 months.

### Design of the GL-Geoformer

Despite remarkable advances in DL for weather forecasting in meteorology (*35*–*38*), few DL models have been specifically designed for climate systems in terms of 3D multivariate predictions in the global tropics. Most existing DL approaches for ENSO predictions have not taken into account interbasin interactions, presenting a gap in DL-based climate modeling. To address this gap, we develop GL-Geoformer, a DL-based global tropical model designed to simulate interannual variability across the coupled ocean-atmosphere system.

Specifically, the model architecture adopts a Vision Transformer framework (*39*–*41*), which is structured on an encoder-decoder configuration, wherein all modules except for the preprocessing component are constructed using self-attention mechanisms (detailed architecture in fig. S14). The complete architecture comprises four principal components: an input data preprocessing module, encoder, decoder, and output layer, with both the encoder and decoder containing three stacked submodules. The preprocessing module initially extracts neighborhood spatial information from high-resolution fields through convolutional operations, in which a set of 3 × 4 convolutional kernels process multichannel spatial information and encode it into 160-dimensional patch embeddings that subsequently receive temporal and spatial positional encodings. The processed input fields are then analyzed through multihead spatiotemporal self-attention mechanisms (set the number of heads to 4) within the encoder architecture, which simultaneously computes temporal correlations across different time steps and spatial relationships among variables. This self-attention approach provides notable advantages over traditional methods by enabling direct, parallel computation of long-range dependencies without the locality constraints inherent in convolutional operations (*42*). Therefore, the GL-Geoformer can dynamically focus on relevant spatiotemporal features regardless of their temporal or spatial distances. This is the primary factor contributing to GL-Geoformer’s exceptional performance.

The decoder generates multivariate predictions using a rolling manner similar to dynamic models. Specifically, in the initial prediction step, the decoder integrates the final month’s multivariate fields from the encoder input predictors with encoder-processed feature maps used to generate predictions for the subsequent month. Subsequent prediction steps recursively incorporate previous predictions alongside encoder feature maps to advance the forecasting sequence. This rolling prediction strategy overcomes limitations of one-step prediction approaches by maintaining physical consistency through continuous incorporation of preceding multimonth information. In this way, multiple oceanic and atmospheric fields representing the inherent coupling relevant to climate mode dynamics are incorporated adequately, thereby facilitating anomaly coupling between the ocean and atmosphere on a month-by-month basis during both the training and predicting phases.

The optimization framework in pretraining stage uses a composite loss function *L* that integrates the RMSE computed across all predicted multivariate fields with additionally a targeted RMSE component specifically calculated for Niño3.4 averaged SST anomalies, ensuring both global accuracy and ENSO prediction precision

$$L=\frac{1}{T_{\text{out}}}\sum_{t=1}^{T_{\text{out}}}\left[\mathrm{SE}\left(Y_{t}^{\prime },Y_{t}\right)+\;\mathrm{MSE}\left(\mathrm{Ni}\tilde{n}o3.4_{t}^{\prime },\mathrm{Ni}\tilde{n}o3.4_{t}\right)\right]$$

where $Y^{\prime }$ is the model multivariate predictions, and *Y* is target predictands; $\mathrm{Ni}\tilde{n}o3\;.\;4^{\prime }$ and $\mathrm{Ni}\tilde{n}o3\;.\;4$ are predicted and target Niño3.4 indices calculated from SST anomaly fields; $T_{\text{out}}$ is the time series length of monthly outputs. To better capture teleconnections and ocean-atmosphere coupling, we propose a coupling-enhanced physical coupling loss (see text S1.3 for details) during transfer training phase. This objective function not only constrains the pixel-wise RMSE of wind and temperature fields but also synchronizes their spatiotemporal covariance.

During model training, we use the Adam optimization algorithm alongside a warm-up learning rate schedule to promote stable convergence. The peak learning rate was set to 1 × 10^−3^ for pretraining and 1 × 10^−5^ for transfer learning, with 3000 and 200 warm-up steps, respectively. In addition, an early stopping technique is implemented to prevent the model from overfitting by terminating training when RMSE loss on the validation set fails to improve for four consecutive epochs during pretraining or three consecutive epochs during transfer learning.

## Supplementary Materials

### This PDF file includes:

Supplementary Texts S1 to S3 Figs. S1 to S14 Tables S1 to S3 References
