# Supplementary material for: Tropical basin interactions reduce spring predictability barrier of ENSO in a deep learning model
Source: Sci Adv. 2026 May 20;12(21):eaeb0901. doi: 10.1126/sciadv.aeb0901 (PMC13189121; doi:10.1126/sciadv.aeb0901)
Supplement: Supplementary file 1 — Supplementary Texts S1 to S3 Figs. S1 to S14 Tables S1 to S3 References [file sciadv.aeb0901_sm.pdf]

Science Advances
AAAS

Supplementary Materials for

Tropical basin interactions reduce spring predictability barrier of ENSO in a deep learning model

Lu Zhou and Rong-Hua Zhang

Corresponding author: Rong-Hua Zhang, rzhang@nuist.edu.cn

Sci. Adv. **12**, eaeb0901 (2026)
DOI: 10.1126/sciadv.aeb0901

This PDF file includes:

Supplementary Texts S1 to S3
Figs. S1 to S14
Tables S1 to S3
References

Supplementary Text

Text S1. The architecture of the GL-Geoformer

The GL-Geoformer adopts an encoder-decoder architecture, comprising two data preprocessing modules, an encoder constructed from stacked attention blocks, a decoder, and an output layer (Fig. S14). The model takes a multivariate sequence $X^{\text{in}} \in \mathbb{R}^{TI\times C\times H\times W}$ spanning TI=9 months as input predictors to predict the evolution of the same variables over a future period of T_{out} months, written as $X^{out} \in \mathbb{R}^{T_{out}\times C\times H\times W}$. At each time step, the input predictors comprise 13 physical variables: zonal wind stress (τ_x), meridional wind stress (τ_y), and 11-layer upper-ocean temperature anomalies at depths of 5, 20, 40, 60, 80, 100, 120, 140, 160, 180, 200 m. These variables are concatenated along the channel dimension (i.e., C=13) and spatially interpolated to a consistent resolution of $H\times W$=75×180 grid points.

To simultaneously capture local physical dependencies and global teleconnection patterns within the input predictors X^{in}, we develop a hybrid architecture comprising a convolutional preprocessing module and a Transformer-based encoder (Figs. S14a-b). Specifically, within the preprocessing module, we implement a single-layer convolutional operation to aggregate local neighborhood information from the input fields. The convolutional kernel size and stride are both configured as $h_0\times w_0$=3×4, with input channel dimensions of C=13 and output channel dimensions of $C\times h_0\times w_0$=13×3×4, respectively. The resulting post-convolution feature map is designated as $X'^{\text{in}} \in \mathbb{R}^{TI\times N\times(C\times h0\times w0)}$, in which N is set to $(H/h_0)\times(W/w_0)$. Subsequently, a linear layer $l(\cdot)$ is used to project X'^{in} into an embedding space of dimension d=160. To preserve the spatiotemporal context, we augment this representation with learnable spatial position encodings S^{en} and sinusoidal temporal position encodings T^{en}. The resulting embedding, $X^{\text{em}}=l(X'^{\text{in}})+S^{\text{en}}+T^{\text{en}} \in \mathbb{R}^{TI\times N\times d}$, serves as the latent representation fed into the subsequent encoder blocks.

S1.1 The encoder

The encoder transforms the latent embedding X^{em} into a sequence of continuous feature representations by leveraging a spatiotemporal attention mechanism (Fig. S14c). The encoder architecture consists of n_1=3 stacked identical blocks, each integrating a multi-head spatiotemporal attention layer (with H_e=4 heads) and a position-wise feed-forward neural network (FNN). To facilitate deep network training and gradient flow, residual connections and

layer normalization are applied after each sub-layer.

For the m_1-th encoding block ($m_1 \leq n_1$), the query ($Q_{m_1}^{\text{en\_t}}$)/key ($K_{m_1}^{\text{en\_t}}$)/value ($V_{m_1}^{\text{en\_t}}$) tensors are derived from the representation $X_{m_1-1}^{\text{en}}$, which is produced by the preceding block (where m_1=1, $X_{m_1-1}^{\text{en}} = X_0^{\text{en}} = X^{\text{em}}$). Specifically, the input is first projected via a fully connected layer $l(\cdot)$ and then partitioned by a function $Cut(\cdot)$ that segments the matrix along the embedding axis into H_e sub-matrices: $Q_{m_1}^{\text{en\_t}}, K_{m_1}^{\text{en\_t}}, V_{m_1}^{\text{en\_t}} = Cut(l(X_{m_1-1}^{\text{en}})) \in \mathbb{R}^{TI \times N \times H_e \times d_h}$. Consequently, the latent dimensionality for each individual attention head is established as $d_h = d/H_e$.

The encoder extracts multi-scale dependencies through a spatiotemporal attention mechanism. First, temporal self-attention is performed to capture the evolution of each spatial patch across the TI months. For each spatial patch n and attention head h, the attention score is computed using a scaled dot-product function: $Atten_{(m_1,h,n)}^{\text{en\_t}} = \text{Softmax}(\frac{Q_{(m_1,h,n)}^{\text{en\_t}} \cdot (K_{(m_1,h,n)}^{\text{en\_t}})^{\text{T}}}{\sqrt{d_h}}) \cdot V_{(m_1,h,n)}^{\text{en\_t}}$, where h=1, 2, …, H_e is an index over the multiple head dimension. The resulting temporal features from all heads and patches are concatenated and projected via a linear layer $l(\cdot)$ to yield the temporal attention output $X_{m_1}^{\text{en\_t}} \in \mathbb{R}^{TI \times N \times d}$.

Subsequently, spatial self-attention is applied to model long-range teleconnections between different geographic locations. Similar to the temporal operation, the spatial attention $Atten_{(m_1,h,i)}^{\text{en\_s}}$ is calculated for each time step i (i=1, 2, …, TI). The final output of the spatial sublayer, $X_{m_1}^{\text{en\_s}} \in \mathbb{R}^{TI \times N \times d}$, is obtained by integrating a residual connection and layer normalization (LN): $X_{m_1}^{\text{en\_s}} = LN(l(\underset{i=1}{\overset{TI}{concat}}(\underset{h=1}{\overset{He}{concat}}(Atten_{(m_1,h,i)}^{\text{en\_s}}))) + X_{m_1-1}^{\text{en}})$.

Finally, the representation is processed by an FNN with another residual shortcut: $X_{m_1}^{\text{en}} = LN(FNN(X_{m_1}^{\text{en\_s}}) + X_{m_1}^{\text{en\_s}})$. After n_1 iterations, the encoder yields the encoder memory matrix $X_{n_1}^{\text{en}} \in \mathbb{R}^{TI \times N \times d}$ (abbreviated as X^{en} in the following, which being also known as encoder memory matrix), which encapsulates multivariate spatiotemporal information for the subsequent decoding stage.

S1.2 The decoder and output layer

The decoder is designed to generate the future trajectory X^{out} based on the encoded

spatiotemporal features. It is composed of n_2=3 identical decoding blocks and three sublayers, which are contained in each block (Fig. S14d). Specifically, the first and third sublayers are composed of multi-head spatiotemporal attention modules and an FNN layer. Additionally, a cross-attention layer is inserted between the self-attention and FNN sublayers to bridge the historical context with future predictions. In this layer, the query ($Q_{m_2}^{\text{en\_de}}$) is derived from the decoder's preceding state, while the keys ($K_{m_2}^{\text{en\_de}}$) and values ($V_{m_2}^{\text{en\_de}}$) are sourced directly from the encoder memory matrix X^{en}.

Mathematically, the cross-attention output for the m_2-th block is formulated as $Atten_{(m_2,h,n)}^{\text{en\_de}} = \text{Softmax}(\frac{Q_{(m_2,h,n)}^{\text{en\_de}} \cdot (K_{(m_2,h,n)}^{\text{en\_de}})^{\text{T}}}{\sqrt{d_h}}) \cdot V_{(m_2,h,n)}^{\text{en\_de}}$. This mechanism allows each position in the predicted sequence to adaptively attend to relevant historical precursors across all input months and spatial locations. Similar to the encoder, each sublayer is encapsulated within residual connections and Layer Normalization to ensure robust feature integration.

Following the n_2 decoding iterations, the final refined representation $X_{n_2}^{\text{de}}$ is processed by the output layer to project the latent features back into the physical domain. This is achieved through a final FNN that captures high-level nonlinear mappings, followed by a dimension reconstruction operation, denoted as $Re(\cdot)$. The reconstruction mapping is formulated as $\hat{X}_{T'_{out}} = Re(FNN(X_{n_2}^{\text{de}})) \in \mathbb{R}^{T'_{out} \times C \times H \times W}, T'_{out} \le T_{out}$; $\hat{X}_{T'_{out}}$ denotes the predicted fields for a lead time T'_{out} . Since the model employs a rolling prediction strategy, T'_{out} increments from 1 to the total prediction months, T_{out}. This operation reshapes the d-dimensional tokens into the original spatial resolution ($H \times W$), effectively generating the predicted multivariate sequences for the future T'_{out} months. By restoring the spatiotemporal dimensions, the GL-Geoformer yields a complete evolution of ENSO-related fields across the specified lead-time periods.

S1.3 Model training strategy

The model training consists of two stages: pre-training and transfer optimization. During pre-training, data from 29 CMIP6 models are used as the training set, and data from one model are used as the validation set. Training is early stopped when the validation loss does not decrease for 4 consecutive epochs. Subsequently, the transfer optimization stage is conducted by initializing from the pre-trained model and updating the model parameters, using all CMIP data

as the training set and SODA reanalysis data prior to 1980 as the validation set.

To address the complexities of long-lead multivariate prediction and mitigate the accumulation of errors inherent in recursive modeling, we adopt a novel training strategy termed Progressive Autoregressive Truth Injection (PATI; *43*) to facilitate a robust transition from teacher-forcing to autonomous rolling prediction. Specifically, during the initial phase of pre-training, the model is primarily guided by ground-truth data (predictands) to ensure stable convergence. As training progresses, the reliance on external truth is systematically reduced, compelling the model to utilize its own prior outputs for subsequent time-step predictions.

For example, we implement a probability-based fusion mechanism to regulate the transition: $X_{fusion} = P \bullet X + (1-P) \bullet X'$. Here, X and X' indicate predictand and model predicted fields, and the probability P for injecting ground truth decreases from 1 to 0 over the training epochs. This progressive curriculum allows the model to benefit from high-fidelity data early on while gradually evolving the capacity for fully autoregressive forecasting over the T_{out} months.

To ensure the model captures both large-scale spatial patterns and specific climate indices, we employ a multi-objective loss function. The loss integrates the RMSE of the multivariate spatial fields with the accuracy of the Niño3.4 index, providing a comprehensive constraint on ENSO evolution. Optimization is performed using the Adam optimizer, supplemented by a learning rate warm-up strategy and an early stopping criterion (with a patience of 4 epochs) to prevent overfitting. Furthermore, to enhance the robustness of our results and quantify uncertainty, we generate an ensemble of ten realizations initialized with different random weights while maintaining a consistent architecture.

During the pre-training phase, the model is trained and validated exclusively using CMIP6 simulations; consequently, systematic biases inherent in these climate models may limit the GL-Geoformer predictive performance. To mitigate this issue, we employ SODA reanalysis data as the validation set during the transfer learning and optimization phase. Simultaneously, a novel Physical Coupling Loss (PCL) is introduced to enhance the GL-Geoformer's representation of air-sea interactions:

$$PCL = L_{weighted} + \lambda_c \cdot L_{coupling},$$

where $L_{weighted} = w_w \cdot MSE(\tau', \tau) + w_s \cdot MSE(S', S) + w_{Te} \cdot MSE(Te', Te)$,

$$L_{coupling} = \left| \phi(\tau', S') - \phi(\tau, S) \right|,$$

$$\phi(\tau',S')=\frac{1}{N_{ts}}\sum(\tau'_{t,i,j}\cdot S'_{t,i,j}),\ \ \phi(\tau,S)=\frac{1}{N_{ts}}\sum(\tau_{t,i,j}\cdot S_{t,i,j}).$$

The total loss *PCL* is defined as the sum of the multi-variable weighted MSE loss $L_{weighted}$ and the air-sea coupling loss $L_{coupling}$, where λ_c denotes the coupling intensity coefficient, set to 1. The parameters w_W, w_S, and w_{Te} represent the weights for the MSE of wind stress (τ), SST (S), and subsurface temperature (Te), respectively, and are set to 1.5, 1.5, and 1. Here, ϕ serves as the coupling intensity operator, and N_{ts} denotes the total number of grid points across both temporal and spatial dimensions. The variables τ', S', and Te' represent the predicted values, while τ, S, and Te denote the ground truth.

During the transfer optimization phase, the GL-Geoformer performs iterative rolling predicting based entirely on its own prior outputs. In this stage, the encoder parameters are frozen, and only the decoder parameters are updated. The learning rate is reduced to 1×10^{-5}. The early stopping criterion is defined as the point where the *PCL* on the SODA validation set fails to decrease for three consecutive epochs. Furthermore, during the training process, this rolling prediction training approach requires preserving gradient information from each step, resulting in substantial memory consumption. Constrained by GPU memory limitations, we set T_{out}=18 in the pre-training stage, T_{out}=9 in the transfer learning stage, and in the T_{out}=24 in the testing phase.

Text S2. Interpretability Analyses using an Integrated Gradient method

Recent advancements in explainable artificial intelligence (XAI) methods have helped uncover internal behaviors of DL models, which are traditionally considered as black-box models. In this section, we utilize a specific XAI approach based on integrated gradients (IGs; *44*) to identify precursor signals in the input predictors. The IG method aims to quantify the contribution of each input feature to GL-Geoformer's predictions, thereby revealing how regional variables affect the model predictions. The IG-based analysis provides an additional validation for the physical mechanisms discussed previously.

The IG method operates by establishing a baseline (X_{base}^{in}), which serves as a neutral reference point in the input space. In this study, the baseline is defined as a random noise matrix (uniformly distributed within [-0.2, 0.2]) with the same dimensions as the input X^{in}. The attribution is calculated by integrating the gradients of the model's output with respect to the input along a straight-line path from the baseline to the actual input predictors.

To implement this numerically, the integral is approximated through Ns=50 discrete interpolation steps. For each step k, the interpolated input X_k^{in} is defined as $X_k^{in} = X_{base}^{in} + \frac{k}{N_s}(X^{in} - X_{base}^{in})$. Consequently, the integrated gradient for each input feature i is formulated as $IG(X_i^{in}) = (X_i^{in} - X_{base,i}^{in}) \times \sum_{k=1}^{N_s} \frac{\partial F(X_k^{in})}{\partial X_i^{in}} \times \frac{1}{N_s}$.

This process yields a set of attribution values for each input feature. A higher absolute attribution value indicates that the corresponding physical variable or geographic region exerts a more dominant influence on the model's prediction. By visualizing these attributions, we can provide an additional level of validation, ensuring that the model's internal logic aligns with established physical mechanisms of climate variability. Here, we compute IG-derived saliency values for ocean temperature predictors spanning 9 consecutive months (July 2019-March 2020), which are used for predictions initiated in April 2020. As previously noted, a crucial factor enabling GL-Geoformer to accurately predict the 2020 La Niña event stems from its capacity to incorporate multivariate conditions from consecutive months into the initial conditions, thereby simultaneously accounting for the interbasin relay effects on central-eastern Pacific SST variability. The IG-based analysis results support this hypothesis (Fig. S12): For instance, during autumn 2019, a strong positive IOD event emerged in the tropical Indian Ocean, which likely

influenced the subsequent La Niña in winter 2020. Correspondingly, the IG analysis reveals high saliency values of temperature predictors over the equatorial Indian and Pacific Oceans, while the Atlantic shows minimal signals (Figs. S12a-f). In the Indian Ocean, temperature sensitivity extends from the surface to 200 m depth, indicating that both surface and subsurface temperature anomalies during autumn 2019 contributed to the 2020-2021 La Niña. Meanwhile, Pacific temperature sensitivity shifts westward from the central-eastern to the western Pacific with increasing depth, reflecting thermocline structure and highlighting the role of thermocline feedback processes. Saliency patterns in winter 2019 (Figs. S12g-l) and spring 2020 (Figs. S12m-r) differ markedly from those in autumn 2019: the Atlantic surface exhibits pronounced high-sensitivity singles, which suggests that the Atlantic SST warming during this period primarily influences 2020-2021 La Niña. Concurrently, Indian Ocean temperature sensitivity concentrates between 15°N and 15°S, extending from surface to subsurface layers, while Pacific temperature sensitivity distributes along thermocline depths, concentrating at the surface in the central-eastern Pacific and at depth in the western Pacific.

These model-based interpretability findings are consistent with the physics-based mechanism analysis and demonstrate the varying contributions of different ocean basins to the 2020-2021 La Niña event. Notably, contributions from the Indian and Atlantic Oceans are comparable to those from the Pacific, further emphasizing the need for multi-basin modeling in tropical climate predictions.

Text S3. Individual and combined effects of tropical basin interactions

S3.1 Full-basin Prediction

Figures 4**A**,**I** illustrate the results from the control experiment ($E_{IndPacAtl}$). Note that most previous physics-driven climate models initialized in spring of 2020 systematically underestimate the intensity of this event, with predicted Niño3.4 SST anomaly amplitudes reaching only about one-third of the observations (Fig. S10). The $E_{IndPacAtl}$ experiment shows predictions which are compared with observations and physics-driven climate models.

It is worth noting that there is apparent difference in predictive performance between the GL-Geoformer and traditional climate models. A crucial factor lies in their initialization strategies: in conventional dynamical models, a single instantaneous ocean-atmosphere state is used for initialization. In contrast, the GL-Geoformer incorporates multi-month input fields as initial conditions for each-step (month) prediction. This temporal incorporation capability enables the DL model to better capture evolving anomaly patterns and their cumulative effects on subsequent evolution. Although dynamical models can use data assimilation techniques to integrate past information for future predictions, this represents an indirect approach that proves substantially less effective than the GL-Geoformer, in which the multi-month temporal data are directly utilized as initial conditions. For example, observations indicate that the strong positive IOD event during September-November 2019, followed by basin-wide Indian Ocean warming and concurrent positive Atlantic SST anomalies during 2020 spring (Figs. S9a-c), may serve as important precursors to the subsequent La Niña event (*45*). These distinct and persistent interbasin signals are explicitly incorporated into the GL-Geoformer's multi-month input predictors, enabling the model to account for their delayed remote effects on Pacific climate conditions. The Transformer-based architecture facilitates effective representations of multivariate dependencies and teleconnection patterns, remotely linking Indian Ocean and Atlantic anomalies to the emergence of cold SST anomalies in the eastern tropical Pacific. In this way, this comprehensive representations of interbasin interactions allow the model to skillfully predict both the onset timing and intensity of the 2020-2021 La Niña event associated with the challenging spring-time barrier predictability issues.

S3.2 Pacific-Only Prediction

When both Indian and Atlantic Ocean influences are removed from the model input fields,

the GL-Geoformer largely fails to predict the 2020-2021 La Niña event (Figs. 4**B**,**J**). Under these conditions, the predicted Niño3.4 SST anomaly remains near-neutral in winter 2020 with substantial prediction uncertainty (indicated by ensemble spread in Fig. 4**J**). These degraded predictions mirror the performance of conventional dynamical and statistical models, which similarly underestimate the La Niña strength (Fig. S10). Specifically, the GL-Geoformer without the effects from the Indian and Atlantic Oceans incorrectly produces westerly wind anomalies in the central-western Pacific region, contrasting with easterly anomalies that are observed (Fig. 4**F**; Figs. 5**D**-**F**). This systematic bias disrupts the essential ocean-atmosphere coupling mechanisms required for cold SST anomaly to develop in the central-eastern Pacific, consequently leading to significant underestimation of La Niña intensity in late 2020.

These results clearly suggest that interbasin interactions from the Indian and Atlantic Oceans play a critical role in the initiation and development of the 2020-2021 La Niña event. Additional sensitivity experiments are conducted to elucidate the specific processes through which Indian and Atlantic Ocean variability influences this La Niña event and to quantify their respective contributions to the overall predictive skill as follows.

S3.3 The Role of the Indian Ocean

E_{IndPac} demonstrates a markedly improved predictive skill relative to E_{Pac} when Indian-Pacific interactions are incorporated in GL-Geoformer predictors (Figs. 4**C**,**K**). In this configuration, the predicted Niño3.4 SST anomaly reaches -0.75°C in winter 2020, achieving nearly 60% of the intensity predicted by the control experiment while still showing substantially reduced prediction uncertainty (shading in Fig. 4**K**). Further mechanistic analysis reveals that the improved prediction stems from coupled atmospheric and oceanic teleconnection pathways: the extreme 2019 IOD event and subsequent basin-wide Indian Ocean warming during spring 2020 induce persistent easterly wind anomalies over the central-eastern Pacific through the Walker circulation adjustments (Figs. S11a-c; Figs. S9c-e), which enhances the Bjerknes feedback, thereby amplifying cold SST anomaly development in the eastern Pacific (Figs. 4**C**,**G**).

Simultaneously, subsurface warm temperature anomalies in the eastern Indian Ocean propagate eastward via equatorial Kelvin waves through Indonesian Through Flow (ITF), causing thermocline deepening in the western Pacific and corresponding shoaling in the eastern Pacific (Figs. S11a-c). This oceanic adjustment promotes enhanced upwelling of subsurface cold

waters in the eastern Pacific, which then interact with surface wind anomalies through the Bjerknes feedback mechanism, further amplifying the cold SST anomalies in the eastern Pacific (Figs. 4**G-I**). Notably, when Indian Ocean climatic conditions are included in the GL-Geoformer, the predicted subsurface temperature anomalies in the tropical Pacific get stronger significantly (exceeding 1.5°C enhancement), whereas changes in surface wind stress anomalies remain relatively modest (Figs. S11a-c). These results indicate that Indo-Pacific oceanic processes constitute the dominant forcing mechanism for this La Niña development, although ocean-atmosphere coupling effects remain non-negligible.

S3.4 The Role of the Atlantic Ocean

Next, we examine the effects from the Atlantic Ocean. In contrast to the tropical Indian Ocean, the tropical Atlantic exhibits a less contribution to the 2020-2021 La Niña development (Table S2). Incorporation of Atlantic multivariate fields in the GL-Geoformer yields modest improvements in prediction skill relative to E_{Pac}, with enhanced easterly winds over the central-eastern Pacific and Niño3.4 SST anomalies reaching -0.4°C (approximately 30% of the control experiment intensity; Figs. 4**D**,**H**,**L**). The enhanced Atlantic-Pacific SST gradient strengthens the Atlantic Walker circulation, promoting subsidence over the eastern Pacific and moderately intensifying surface easterly wind stress in the central Pacific (Figs. 5**J**-**L**). However, this mechanism produces only weak wind anomalies that are statistically insignificant across most regions. Consequently, these wind anomalies are not sufficiently strong to drive obvious upwelling in the eastern equatorial Pacific, and subsurface cold temperature anomalies in the central Pacific fail to exhibit coherent eastward propagation along the thermocline (Figs. S11d-f).

S3.5 Synergistic Tropical Basin Effects

These experiments indicate that the strong 2020-2021 La Niña results from simultaneous modulation by both Indian and Atlantic Ocean processes. Previous studies demonstrates that simultaneous warm SST anomalies in the equatorial central-eastern Pacific and equatorial Atlantic (as observed during spring 2020) are able to initially induce Indian Ocean warming through the Tropospheric Temperature mechanism (*46, 47*), which subsequently generates easterly wind anomalies over the western Pacific and then triggers La Niña conditions several months later. However, E_{PacAtl}, in which Indian Ocean effects are excluded, may consequently

underestimate Atlantic contributions to the La Niña development. On the other hand, previous researches indicate that the winter tropical Atlantic acts as a crucial mediator connecting autumn IOD events with subsequent ENSO evolution (*48*), suggesting that positive IOD conditions can be effectively used to predict subsequent La Niña events. These findings emphasize the complex forcing and feedback mechanisms operating across the Indian, Atlantic, and Pacific Oceans, highlighting the necessity of considering the tropical ocean-atmosphere interactions as an integrated system.

In our study, these basin interactions are well captured in the control experiment, with the GL-Geoformer incorporating climatic information from all three tropical basins so that the 2020-2021 La Niña event can be accurately predicted when the model is initiated in April 2020 (Figs. 4**A**,**I**; Fig. S13), a time when tradition models typically suffer from the SPB. As illustrated in Figs. S11g-i, synergistic effects from Indian and Atlantic Ocean anomalies on the Pacific yield stronger surface wind stress and subsurface temperature anomalies than the linear sum of their individual influences. So, it is evident that Indian and Atlantic Ocean influences on Pacific variability exhibit nonlinear rather than simply additive characteristics. Clearly, based on these analyses, this study provides a DL-based quantification of such basin interactions affecting ENSO evolution, highlighting the critical importance of three-ocean interactions and cross-basin processes for ENSO development and prediction (as shown in the schematic diagram Fig. S13).

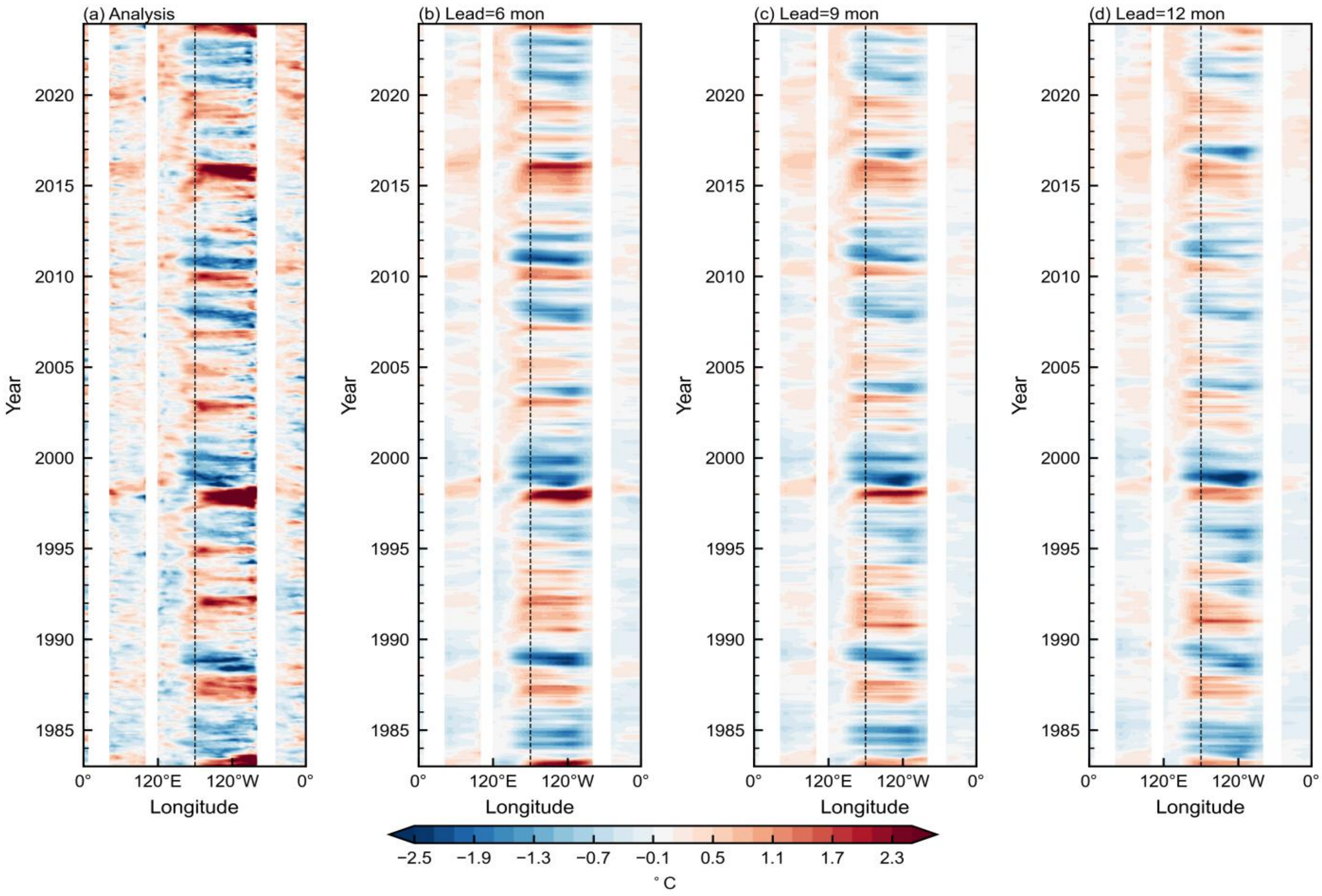

Fig. S1 Evolution of SST anomalies along the equator during 1983-2023. (a) GODAS reanalysis; (b-d) the GL-Geoformer predictions made at 6-, 9-, and 12-month lead times, respectively.

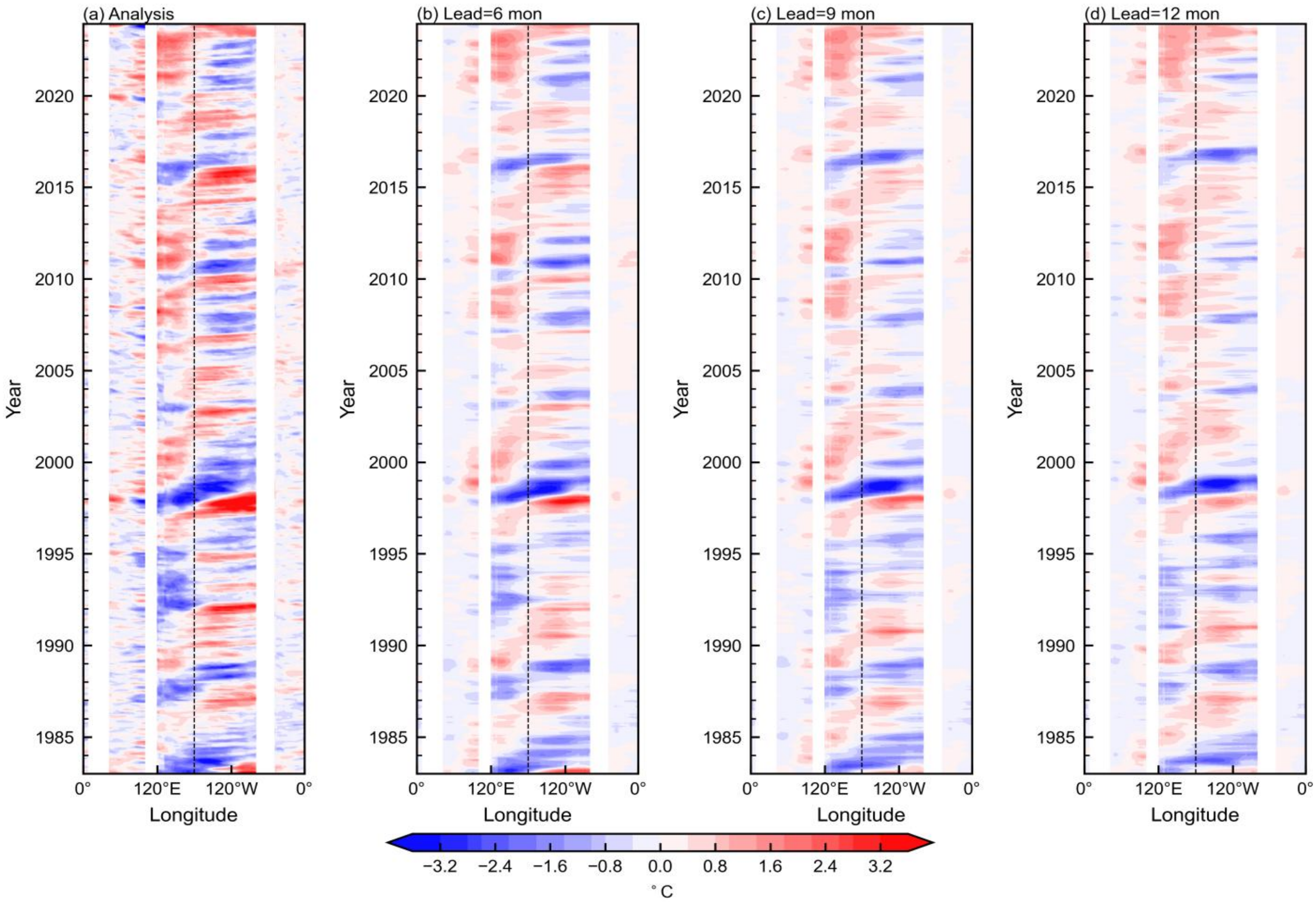

Fig. S2 Same as Fig. S1, but for the averaged temperature anomalies of the upper 200 m (T200).

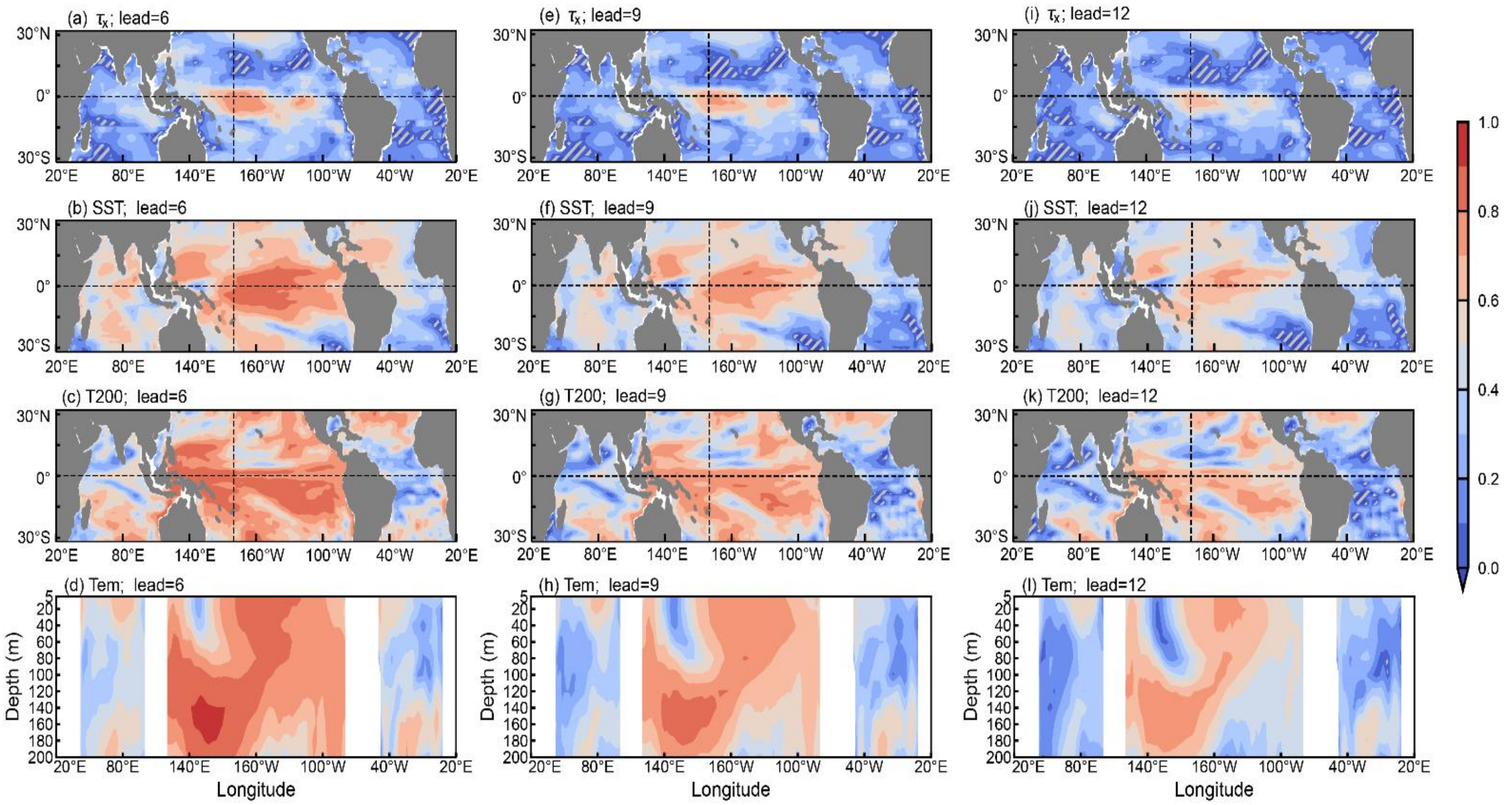

Fig. S3 Spatial distributions of ACCs calculated between GODAS reanalysis and model predictions during 1983-2023. The results assessed at lead times of (a-d) 6-month, (e-h) 9-month, and (i-l) 12-month are displayed for τ_x, SST, T200 and ocean temperature anomalies in the zonal-depth sections on the equator, respectively. The hatched region indicates the ACCs are below 95% confidence level based on Student's t test.

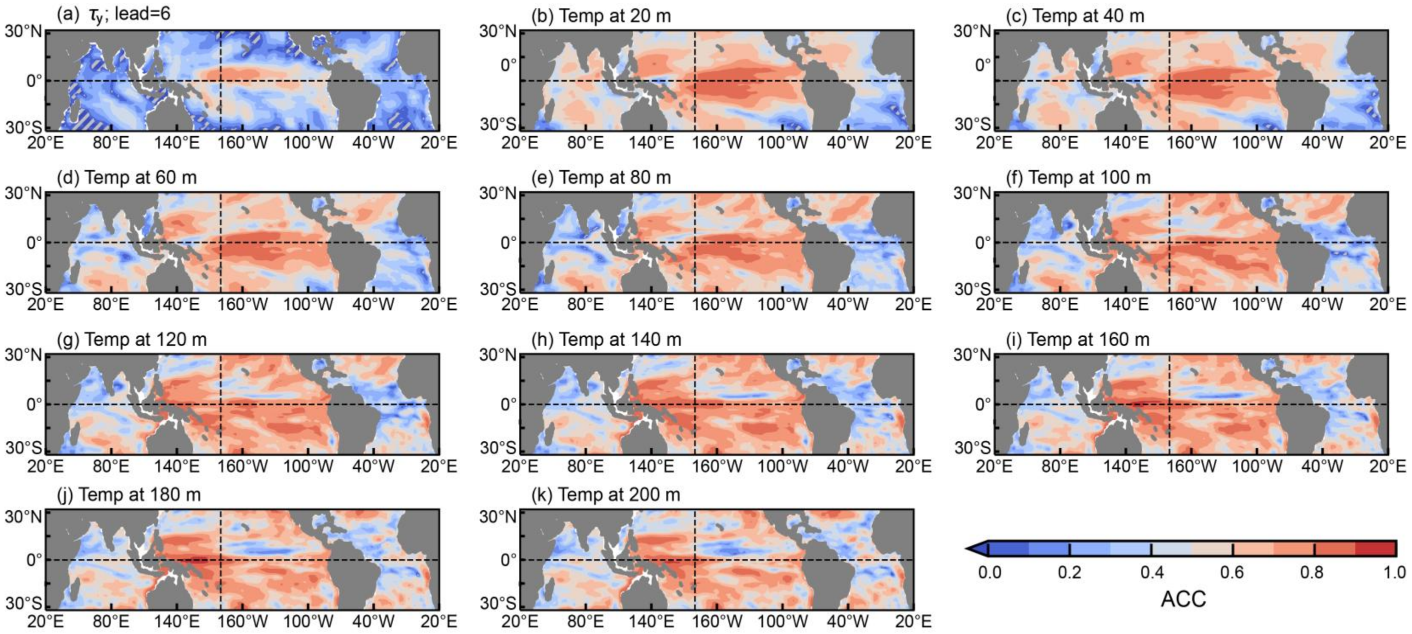

Fig. S4 Distributions of multivariate ACCs in reanalysis and predictions made using GL-Geoformer at a 6-month lead time during period 1983-2023. Results are shown for (a) τ_y and (b-i) upper ocean temperature anomalies at depths of 20, 40, 60, 80, 100, 120, 140, 160, 180, 200 m. The shading indicates region where ACCs are below 95% confidence level based on Student's t test.

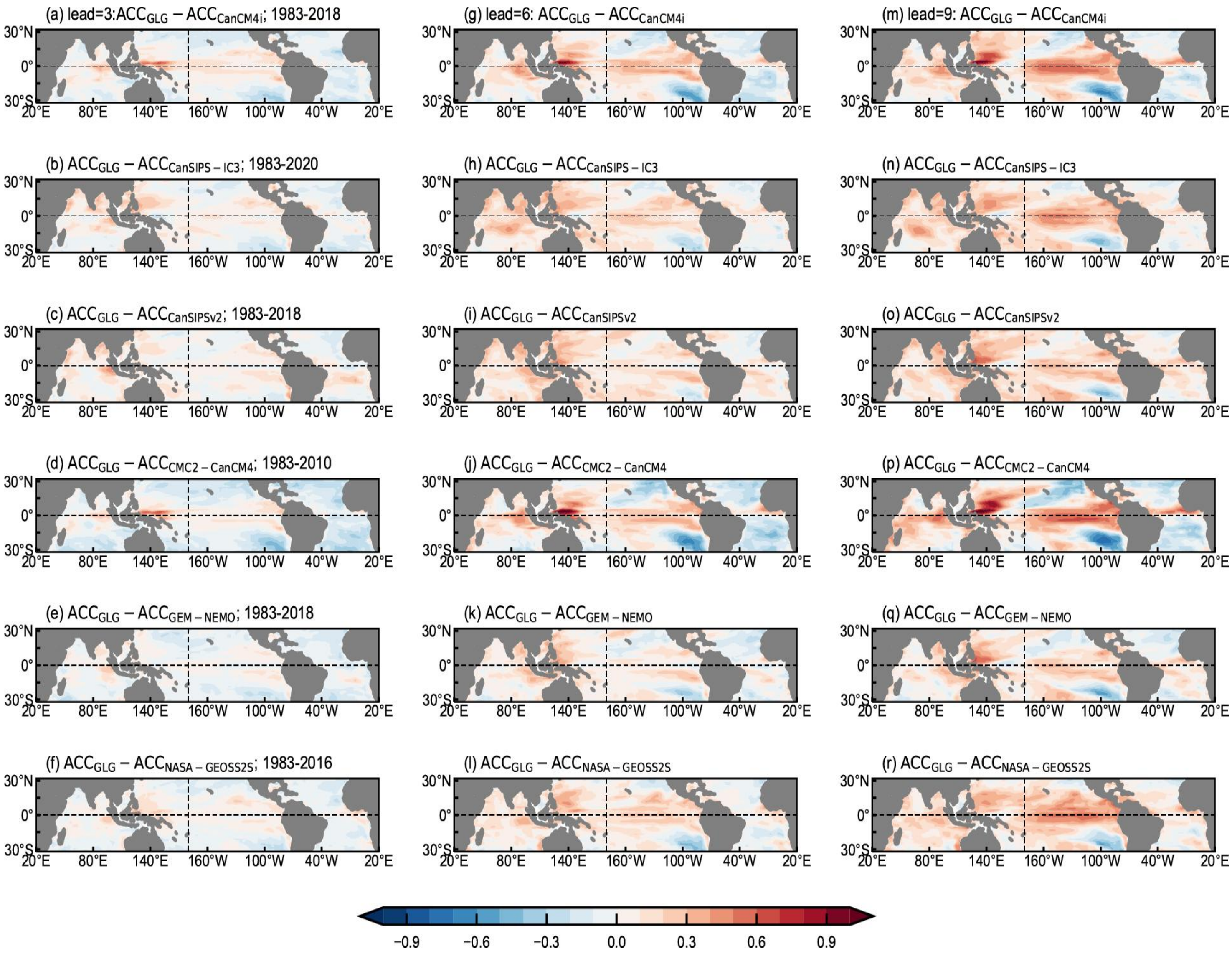

Fig. S5 Distributions of the ACC differences between the GL-Geoformer and climate models from the NMME products. The prediction ACC skills are assessed at lead times of 3-, 6- and 9-month (the first, second and third columns), respectively. The red shadings indicate regions where the GL-Geoformer outperforms the dynamical models in the NMME.

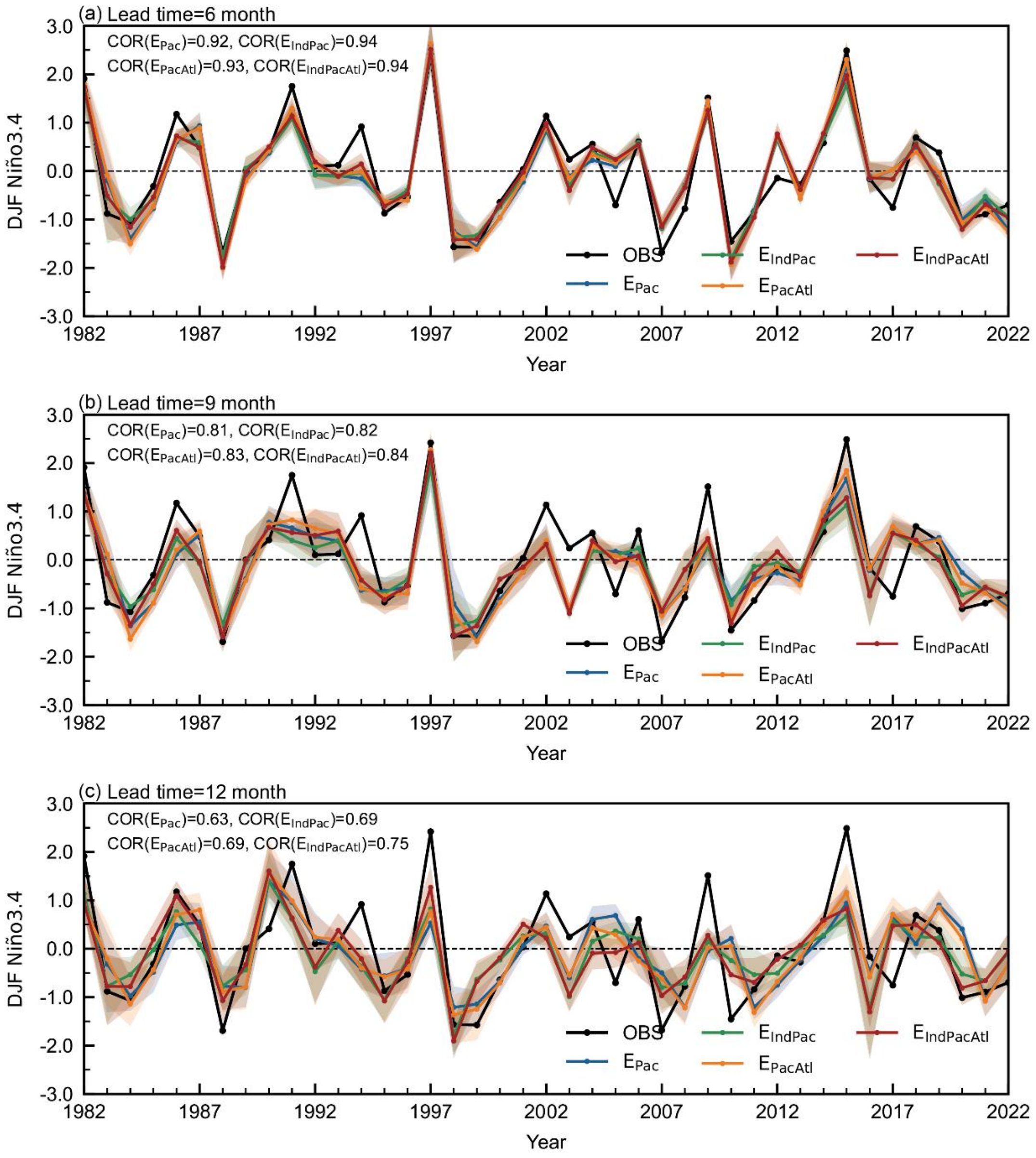

Fig. S6 Time series of DJF season Niño3.4 SST anomalies for (a) 6-, (b) 9-, and (c) 12-month-lead predictions made using the GL-Geoformer in the $E_{IndPacAtl}$ (red), E_{Pac} (blue), E_{IndPac} (green), and E_{PacAtl} (orange) experiments.

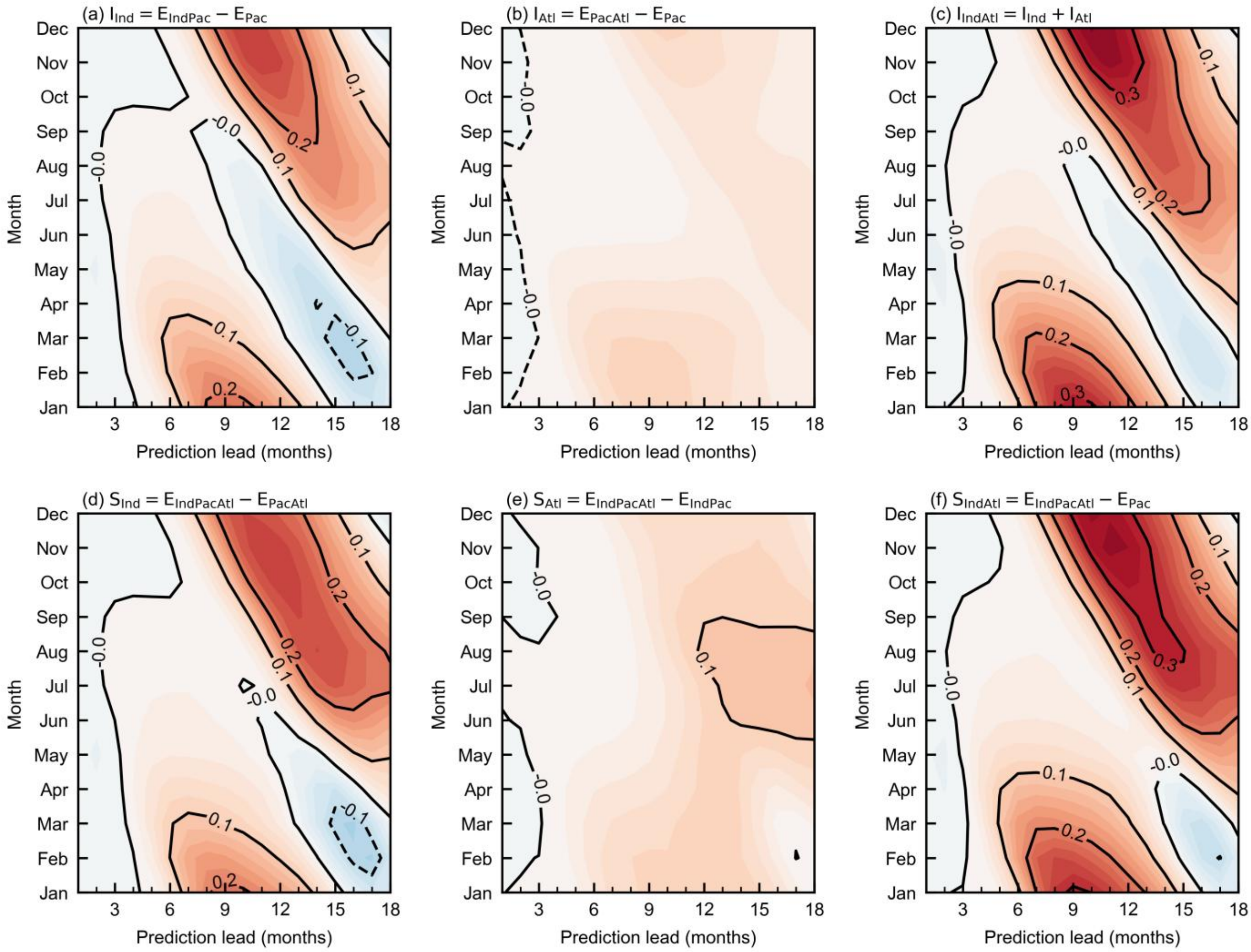

Fig. S7 Individual and synergistic influences of the Indian and Atlantic Oceans on ENSO predictability assessed during 1983-2023. (a-b) Seasonal distribution of individual contributions to Niño3.4 correlation skill from the Indian Ocean (I_{Ind}) and Atlantic Ocean (I_{Atl}) as a function of lead time and calendar month. I_{Ind} is defined as E_{IndPac}-E_{Pac}, and I_{Atl} as E_{PacAtl}-E_{Pac}, representing cases where the other non-Pacific basin is masked. (c) The linear summation of the individual influences from Indian Ocean and Atlantic Ocean. (d-e) Marginal contributions of the Indian Ocean and Atlantic Ocean within the fully coupled three-basin system. The Indian Ocean contribution (S_{Ind}) is derived as $E_{IndPacAtl}$-E_{PacAtl}, and the Atlantic Ocean contribution (S_{Atl}) is derived as $E_{IndPacAtl}$-E_{IndPac}, implicitly accounting for the presence of the other basin effects. (f) The total synergistic influence (S_{IndAtl}) obtained from $E_{IndPacAtl}$-E_{Pac}, representing the aggregate gain from synchronized global interbasin interactions (also see Table S1).

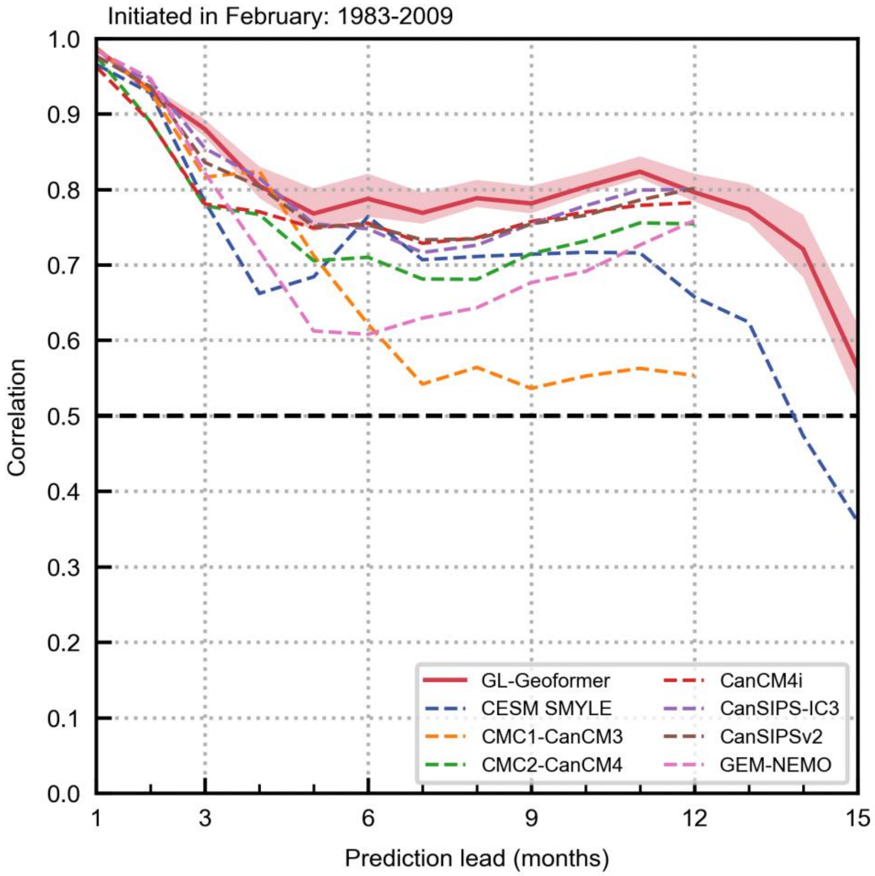

Fig. S8 Correlation skill of Niño3.4 SST anomaly predictions initiated in February as a function of lead month. The solid red line denotes the ensemble-mean skill of the GL-Geoformer, and the dotted lines represent the ensemble-mean skills of physics-based climate models from NMME and CESM SMYLE products. The evaluation period is 1983-2009, and shading indicates the 95% confidence interval estimated using bootstrap resampling.

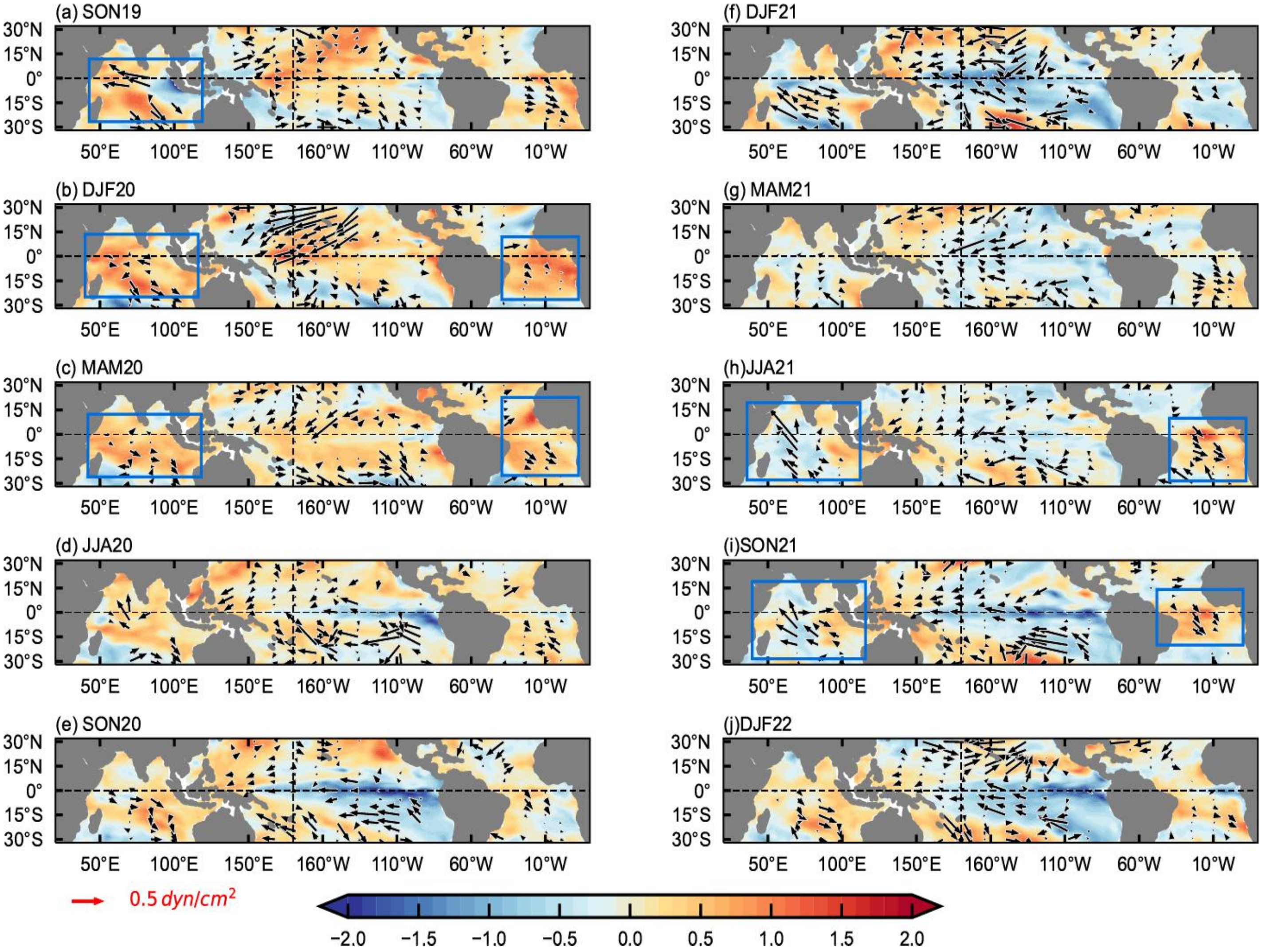

Fig. S9 Seasonal-mean evolutions of GODAS reanalyzed wind stress (vectors) and SST (shading; ℃) during SON 2019-DJF 2022.

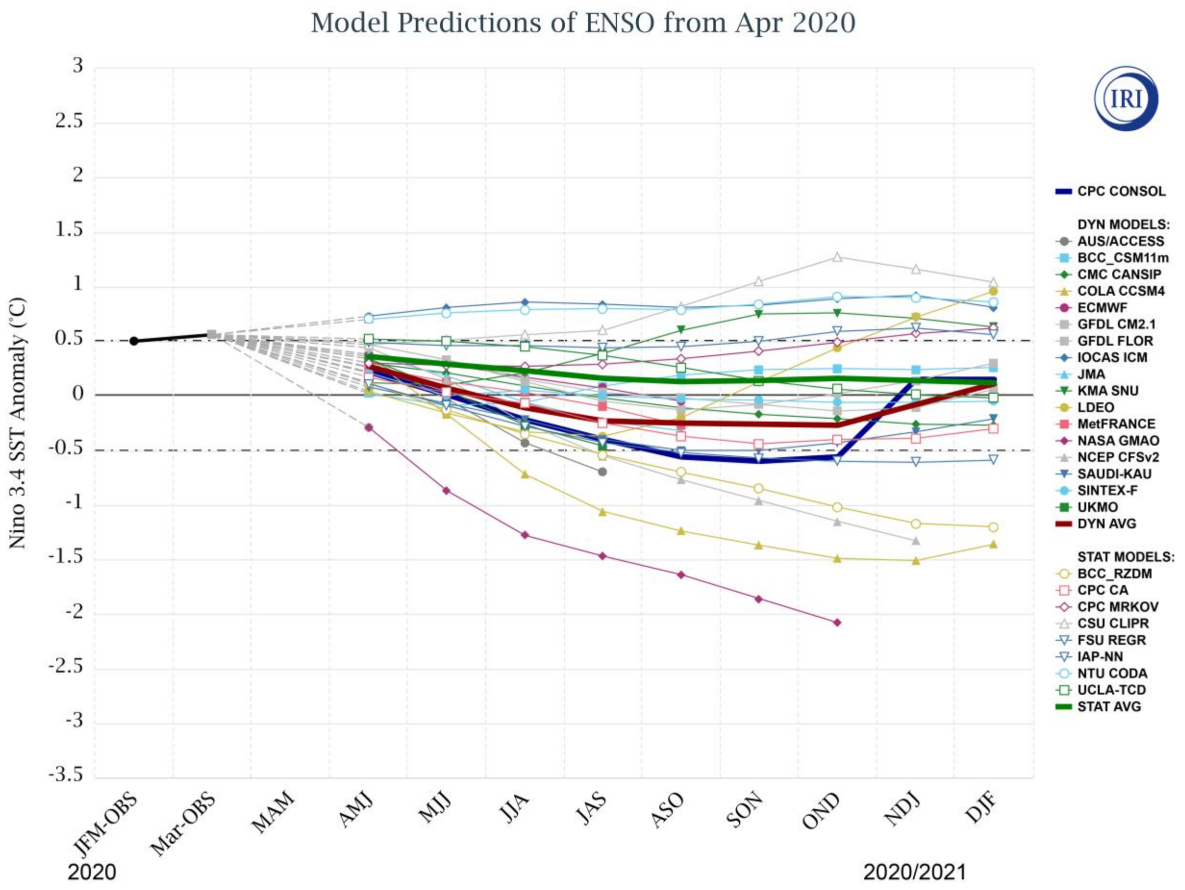

Fig. S10 The Niño3.4 SST anomalies in 2020-2021 predicted (colored lines) in mid-April 2020 using different models. Each colored line indicates a 3-month running mean of 12-month prediction. Images provided by The International Research Institute for Climate and Society, Columbia University Climate School (https://iri.columbia.edu/ENSO; *12*) and shared under a CC BY 4.0 license.

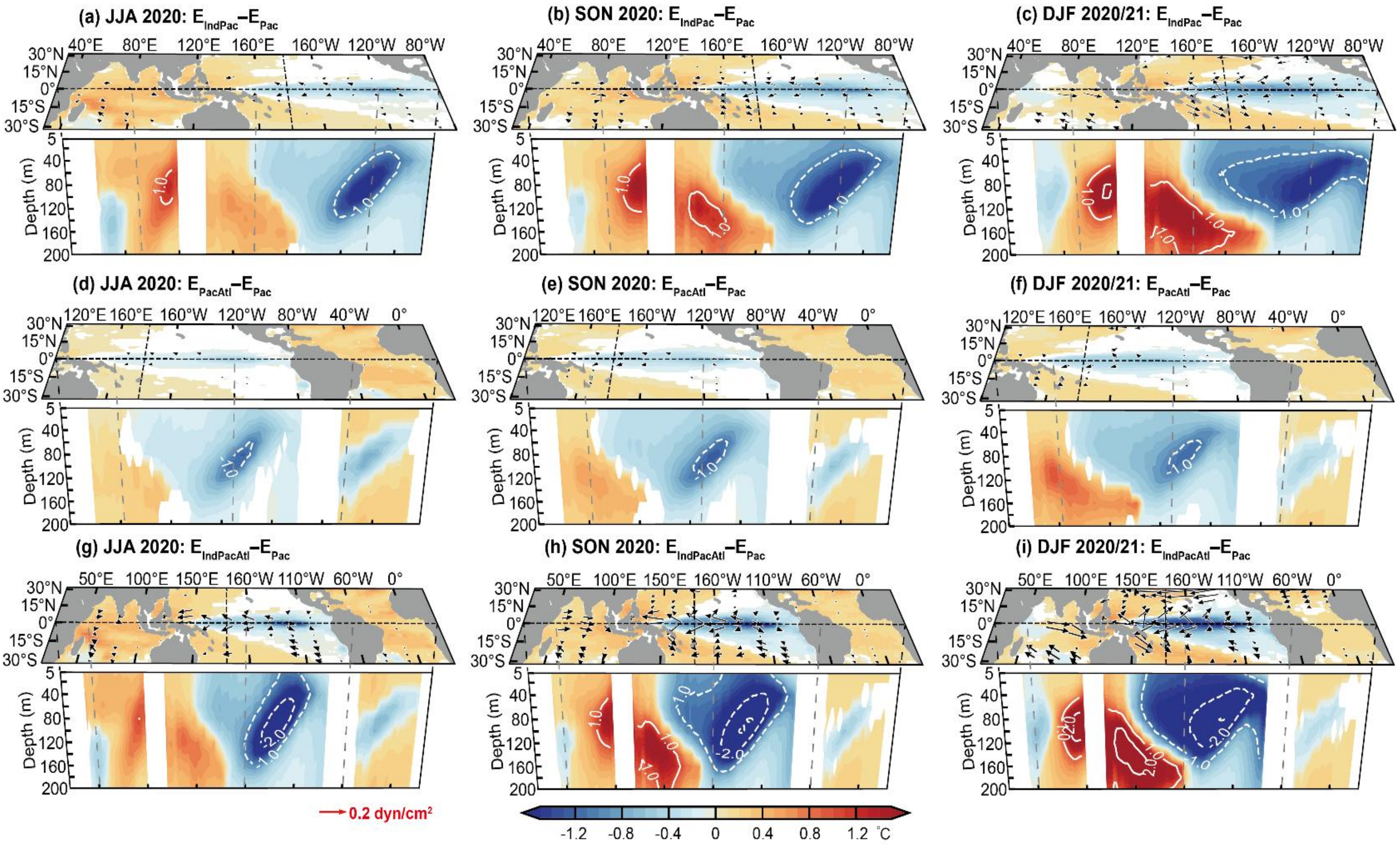

Fig. S11 Effects of Indian and Atlantic Oceans on the 2020-2021 La Niña predictions examined using GL-Geoformer. Figures compare predictions initiated in April 2020 and also show the differences between (a-c) E_{IndPac} and E_{Pac}, (d-f) E_{PacAtl} and E_{Pac}, and (g-i) $E_{IndPacAtl}$ and E_{Pac}. Each figure illustrates the spatiotemporal evolution of the differences in predicted wind stress (vectors) and SST anomalies (shading) in horizontal sections (upper the panel), along with the differences in upper-ocean temperature anomalies (shading and contours) in the equatorial vertical-zonal sections (the low panel). Wind vectors and temperature shadings are only displayed in regions with passing the 95% significance test.

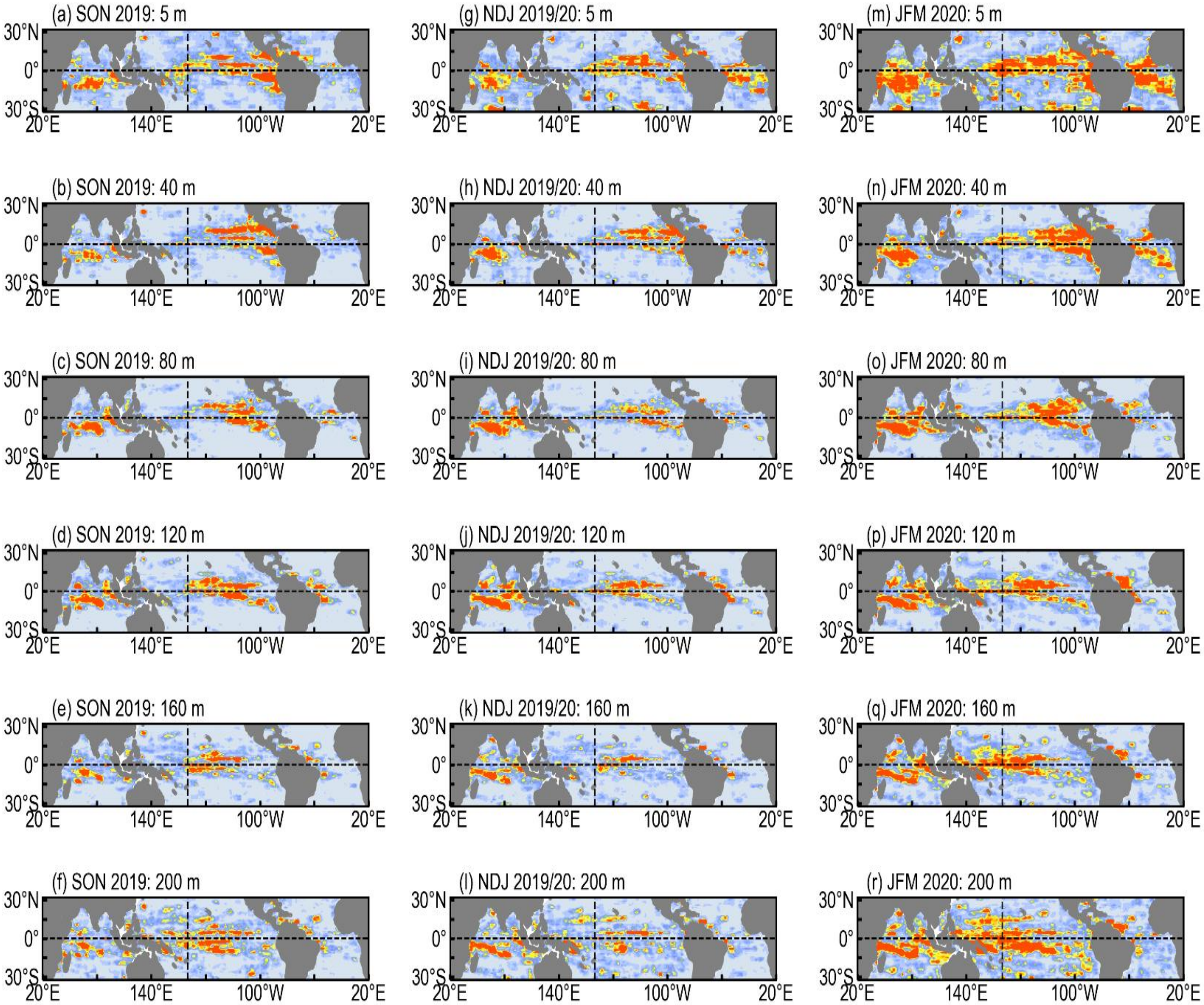

Fig. S12 Saliency analysis of temperature predictors for (a-f) SON 2019, (g-l) DJF 2019/20, and (m-r) JFM 2020, which are employed as initial conditions in GL-Geoformer for predictions initiated from April 2020 to predict the La Niña event in late 2020. Each row presents temperature saliency maps at different depths ranging from 5 to 200 m. Shading indicates saliency values obtained through the integrated gradient method, which highlights the spatial region and the degree to which temperature predictors are crucially important for NDJ 2020-2021 Niño3.4 SST anomaly predictions.

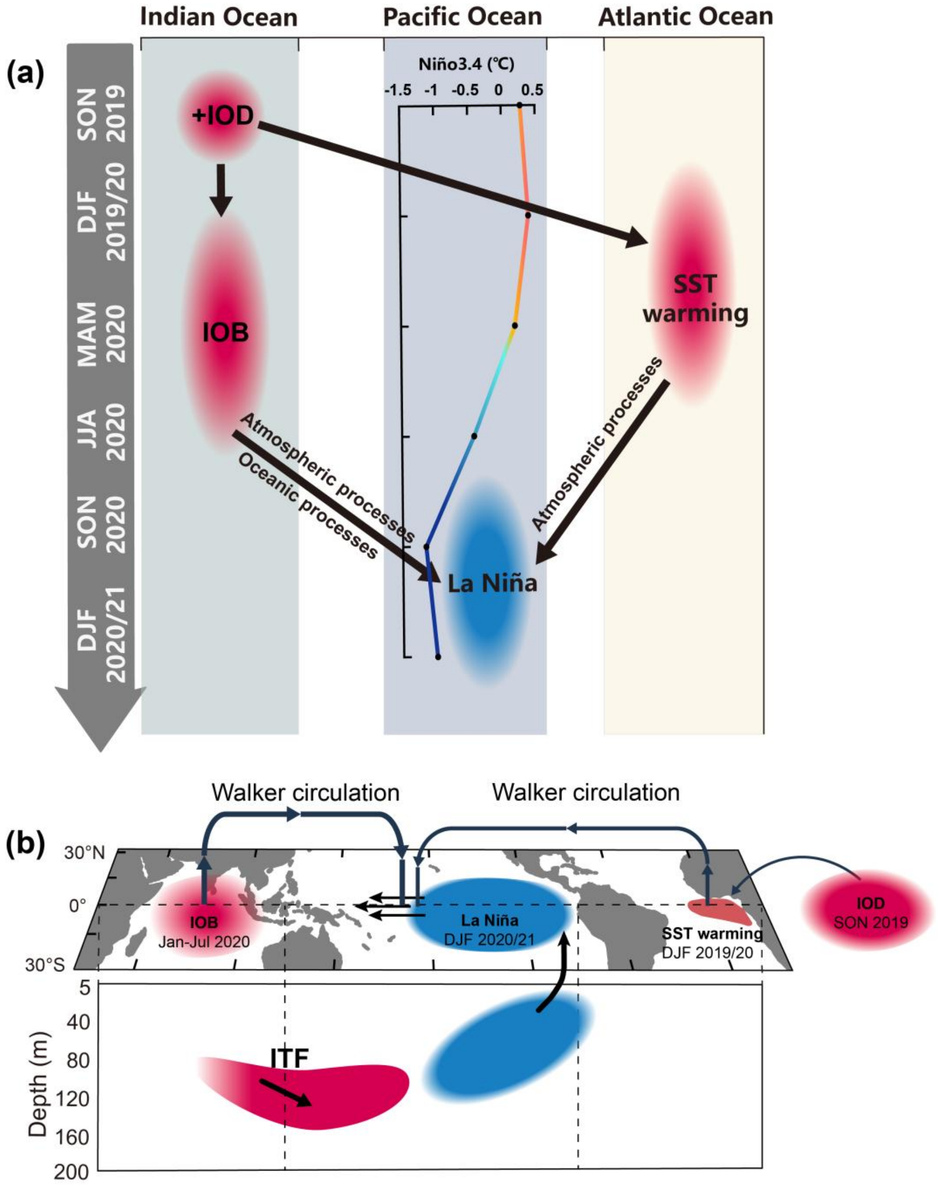

Fig. S13 Schematic diagram showing the 2020-2021 La Niña event mechanism. (a) Illustration for the interbasin synergistic and relay effects on this La Niña development due to the 2019 IOD, 2020 IOB, and 2020 Atlantic SST warming. (b) Detailed schematic showing the physical processes, by which the autumn 2019 IOD induces winter Atlantic Niño-like SST anomalies and the 2020 IOB event. The SST anomalies in both the Indian and Atlantic Oceans enhance the Pacific background wind field through their influence on the Walker Circulation, while Indian Ocean subsurface temperature anomaly signals propagate into the Pacific via the ITF pathway, promoting eastern Pacific subsurface cold water upwelling and thereby facilitating the development of the 2020-2021 La Niña.

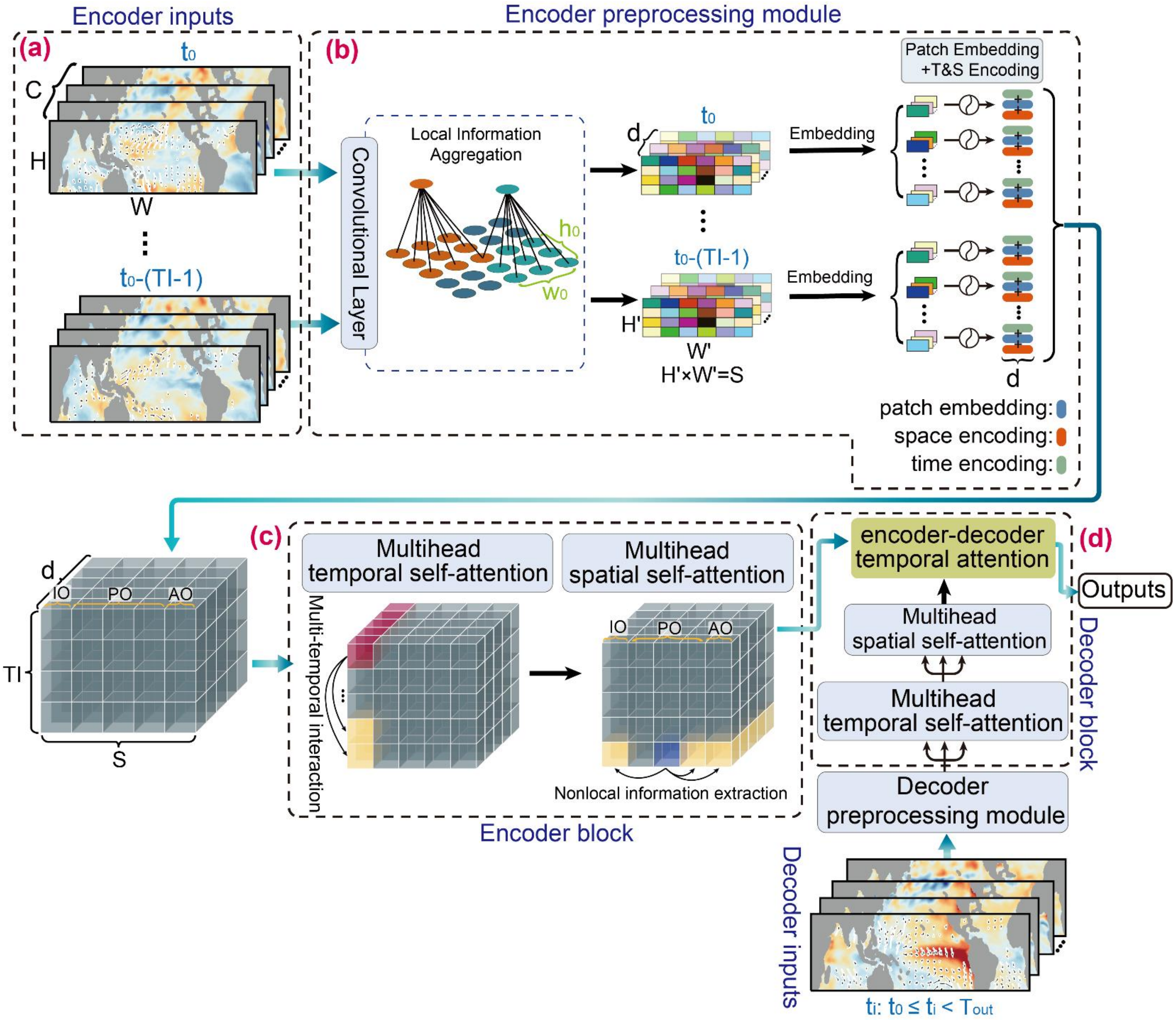

Fig. S14 Architecture of GL-Geoformer for multivariate predictions of the global tropical ocean-atmosphere system. The model employs an encoder-decoder scheme with four key components: two data preprocessing modules, a spatiotemporal self-attention-based encoder, a decoder functioning as a sequence-generation module, and an output layer. (a) The model utilizes two sea surface wind stress components (τ_x and τ_y) and eleven-layer upper ocean temperature anomaly fields as predictors over a finite time interval (*TI*); t_0 denotes the prediction initiation time. (b) The encoder preprocessing module first extracts and encodes local space information from multivariate predictors. Next, it splits the feature maps into S fixed-size non-overlapping patches with shape $h_0 \times w_0$ over the channel dimension (*C*) and embeds them into a dimension of *d*. The

decoder preprocessing module performs identical operations. (c) The encoder processes the embedded matrix through temporal and spatial self-attention analyses. (d) The decoder combines the refined feature maps from the encoder with previously predicted fields as initial conditions to generate predictions for subsequent months.

Table S1. Definition of skill decomposition and their corresponding physical interpretations.

Contribution Category	Calculation Formula	Physical Interpretation
Individual Indian Effect	$I_{Ind}=E_{IndPac}-E_{Pac}$	The solo influence of the Indian Ocean in the Indian-Pacific climate system without Atlantic Ocean interference.
Individual Atlantic Effect	$I_{Atl}=E_{PacAtl}-E_{Pac}$	The solo influence of the Atlantic Ocean in the Pacific-Atlantic climate system without Indian Ocean interference.
Linear Summation	$I_{IndAtl}=I_{Ind}+I_{Atl}=(E_{IndPac}-E_{Pac}) + (E_{PacAtl}-E_{Pac})$	The total effects if the two basins operated independently without any coupling/synergy.
Synergistic Indian Effect	$S_{Ind}=E_{IndPacAtl}-E_{PacAtl}$	The marginal gain from the Indian Ocean when the Atlantic Ocean is already active; including non-linear interactions between basins
Synergistic Atlantic Effect	$S_{Atl}=E_{IndPacAtl}-E_{IndPac}$	The marginal gain from the Atlantic when the Indian Ocean is active.
Synergistic Contribution	$S_{IndAtl}=E_{IndPacAtl}-E_{Pac}$	The combined contribution of the Indian Ocean and Atlantic Ocean in the three-basin coupled system.

Table S2. Niño3.4 SST anomalies (°C) from April 2020 to February 2021 derived from GODAS reanalysis and predicted from various experiments using the GL-Geoformer from the initial conditions in April 2020. The values in parentheses represent the percentage ratio (%) of predicted value relative to observed Niño3.4 SST anomalies, where values closer to 100% indicate higher prediction accuracy. Good results for the predictions and corresponding percentages for each month are indicated in bold formatting.

Data \ Time	2020 Apr	Jun	Aug	Oct	Dec	2021 Feb
GODAS	0.39	-0.12	-0.44	-0.92	-1.26	-1.00
$E_{IndPacAtl}$	0.30(76.9)	-0.22(187.2)	-0.60(136.7)	**-0.93(101.3)**	**-1.21(96.0)**	**-1.13(113.3)**
E_{Pac}	**0.38(97.2)**	0.21(-175.2)	0.13(-30.7)	0.06(-6.8)	0.02(-1.4)	0.05(-4.6)
E_{IndPac}	0.33(84.0)	**-0.08(70.1)**	**-0.37(85.3)**	-0.58(63.0)	-0.77(61.0)	-0.68(67.8)
E_{PacAtl}	0.34(86.3)	0.04(-33.3)	-0.13(29.8)	-0.29(31.4)	-0.40(32.1)	-0.40(40.4)

Table S3. Details of the CMIP6 simulations and reanalysis data used in this study. The CMIP6 and SODA datasets serve as the training and validation sets, and the GODAS dataset is used as the testing set.

Note: As outlined in the paper, the model is trained on CMIP6 historical simulations (1850-2014) and tested on reanalysis (1980-2023). While the periods appear to overlap, this does not introduce bias because the dates in the historical simulations represent "model years" within the simulation framework, which are not actual calendar time.

No.	CMIP6 Model Source	Modeling Group	Variant Label	Period
1	AWI-CM-1-1-MR	AWI	r1i1p1f1	Jan. 1850-Dec. 2014
2	AWI-ESM-1-1-LR	AWI	r1i1p1f1	
3	CanESM5-CanOE	CCCma	r1i1p2f1	
4	CanESM5-1	CCCma	r1i1p1f1	
5	CESM2	NCAR	r10i1p1f1	
6	CESM2-WACCM-FV2	NCAR	r1i1p1f1	
7	CESM2-WACCM	NCAR	r1i1p1f1	
8	CMCC-CM2-HR4	CMCC	r1i1p1f1	
9	CMCC-ESM2	CMCC	r1i1p1f1	
10	CNRM-CM6-1	CNRM-CERFACS	r1i1p1f2	
11	E3SM-1-0	E3SM-Project LLNL UCI UCSB	r1i1p1f1	
12	E3SM-1-1-ECA	E3SM-Project	r1i1p1f1	
13	E3SM-1-1	E3SM-Project RUBISCO	r1i1p1f1	
14	E3SM-2-0	E3SM-Project	r1i1p1f1	
15	E3SM-2-0-NARRM	E3SM-Project	r1i1p1f1	
16	EC-Earth3-CC	EC-Earth-Consortium	r1i1p1f1	
17	EC-Earth3	EC-Earth-Consortium	r1i1p1f1	
18	EC-Earth3-AerChem	EC-Earth-Consortium	r1i1p1f1	
19	EC-Earth3-Veg	EC-Earth-Consortium	r1i1p1f1	
20	EC-Earth3-Veg-LR	EC-Earth-Consortium	r1i1p1f1	
21	FGOALS-g3	CAS	r1i1p1f1	
22	FIO-ESM-2-0	FIO-QLNM	r1i1p1f1	
23	GFDL-CM4	NOAA-GFDL	r1i1p1f1	
24	GFDL-ESM4	NOAA-GFDL	r1i1p1f1	
25	HadGEM3-GC31-LL	MOHC NERC	r1i1p1f3	
26	ICON-ESM-LR	MPI-M	r1i1p1f1	
27	NorCPM1	NCC	r1i1p1f1	
28	NorESM2-MM	NCC	r1i1p1f1	

29	SAM0-UNICON	SNU	r1i1p1f1
30	UKESM1-1-LL	MOHC NERC NIMS-KMA NIWA	r1i1p1f2

No.	Reanalysis Data	Institution	Period
1	SODA	University of Maryland	Jan. 1871-Dec. 1979
2	GODAS	NCEP	Jan. 1980-Dec. 2023

REFERENCES

1. J. Bjerknes, Atmospheric teleconnections from the equatorial pacific. *Mon. Weather Rev.* **97**, 163–172 (1969).

2. M. J. McPhaden, S. E. Zebiak, M. H. Glantz, ENSO as an integrating concept in earth science. *Science* **314**, 1740–1745 (2006).

3. A. Timmermann, S. I. An, J. S. Kug, F. F. Jin, W. Cai, A. Capotondi, K. M. Cobb, M. Lengaigne, M. J. McPhaden, M. F. Stuecker, K. Stein, A. T. Wittenberg, K. S. Yun, T. Bayr, H. C. Chen, Y. Chikamoto, B. Dewitte, D. Dommenget, P. Grothe, E. Guilyardi, Y. G. Ham, M. Hayashi, S. Ineson, D. Kang, S. Kim, W. Kim, J. Y. Lee, T. Li, J. J. Luo, S. McGregor, Y. Planton, S. Power, H. Rashid, H. L. Ren, A. Santoso, K. Takahashi, A. Todd, G. Wang, G. Wang, R. Xie, W. H. Yang, S. W. Yeh, J. Yoon, E. Zeller, X. Zhang, El Niño-Southern oscillation complexity. *Nature* **559**, 535–545 (2018).

4. C. W. Callahan, J. S. Mankin, Persistent effect of El Niño on global economic growth. *Science* **380**, 1064–1069 (2023).

5. C. Gao, R.-H. Zhang, A mechanism for the generation of a warm SST anomaly in the Western Equatorial Pacific: A pathway perspective. *J. Geophys. Res. Oceans* **128**, e2023JC020119 (2023).

6. R.-H. Zhang, L. M. Rothstein, A. J. Busalacchi, Origin of upper-ocean warming and El Niño change on decadal scales in the tropical Pacific Ocean. *Nature* **391**, 879–883 (1998).

7. M. A. Cane, S. E. Zebiak, S. C. Dolan, Experimental forecasts of El Niño. *Nature* **321**, 827–832 (1986).

8. D. Chen, S. E. Zebiak, A. J. Busalacchi, M. A. Cane, An improved procedure for EI Niño forecasting: Implications for predictability. *Science* **269**, 1699–1702 (1995).

9. R.-H. Zhang, C. Gao, The IOCAS intermediate coupled model (IOCAS ICM) and its real-time predictions of the 2015–2016 El Niño event. *Sci. Bull.* **61**, 1061–1070 (2016).

10. R.-H. Zhang, C. Gao, L. Feng, Recent ENSO evolution and its real-time prediction challenges. *Natl. Sci. Rev.* **9**, nwac052 (2022).

11. J. Zhu, W. Wang, A. Kumar, Y. Liu, D. DeWitt, Assessment of a new global ocean reanalysis in ENSO predictions with NOAA UFS. *Geophys. Res. Lett.* **51**, e2023GL106640 (2024).

12. M. A. Ehsan, M. L. L'Heureux, M. K. Tippett, A. W. Robertson, J. Turmelle, Real-time ENSO forecast skill evaluated over the last two decades, with focus on the onset of ENSO events. *npj Clim. Atmos. Sci.* **7**, 301 (2024).

13. N. S. Keenlyside, H. Ding, M. Latif, Potential of equatorial Atlantic variability to enhance El Niño prediction. *Geophys. Res. Lett.* **40**, 2278–2283 (2013).

14. W. Cai, L. Wu, M. Lengaigne, T. Li, S. McGregor, J. S. Kug, J. Y. Yu, M. F. Stuecker, A. Santoso, X. Li, Y. G. Ham, Y. Chikamoto, B. Ng, M. J. McPhaden, Y. Du, D. Dommenget, F. Jia, J. B. Kajtar, N. Keenlyside, X. Lin, J. J. Luo, M. Martin-Rey, Y. Ruprich-Robert, G. Wang, S. P. Xie, Y. Yang, S. M. Kang, J. Y. Choi, B. Gan, G. I. Kim, C. E. Kim, S. Kim, J. H. Kim, P. Chang, Pantropical climate interactions. *Science* **363**, eaav4236 (2019).

15. C. Z. Wang, Three-ocean interactions and climate variability: A review and perspective. *Clim. Dyn.* **53**, 5119–5136 (2019).

16. H. Fan, C. Wang, S. Yang, G. Zhang, Coupling is key for the tropical Indian and Atlantic oceans to boost super El Niño. *Sci. Adv.* **10**, eadp2281 (2024).

17. A. Hu, I. Richter, Y. Okumura, N. Burls, N. Keenlyside, R. Parfitt, K. Bellomo, A. Bellucci, R. Farneti, A. V. Fedorov, B. S. Ferster, C. He, Q. Li, D. Matei, Unraveling the complexity of global climate dynamics: Interactions among El Niño–Southern oscillation, Atlantic meridional overturning circulation, and tropical basins across different timescales. *Ocean Land Atmos. Res.* **4**, 96 (2025).

18. S. Zhao, F.-F. Jin, M. F. Stuecker, P. R. Thompson, J.-S. Kug, M. J. McPhaden, M. A. Cane, A. T. Wittenberg, W. Cai, Explainable El Niño predictability from climate mode interactions. *Nature* **630**, 891–898 (2024).

19. Y. G. Ham, J. H. Kim, J. J. Luo, Deep learning for multi-year ENSO forecasts. *Nature* **573**, 568–572 (2019).

20. L. Zhou, R.-H. Zhang, A self-attention-based neural network for three-dimensional multivariate modeling and its skillful ENSO predictions. *Sci. Adv.* **9**, eadf2827 (2023).

21. M. F. Stuecker, A. Timmermann, F. F. Jin, Y. Chikamoto, W. Zhang, A. T. Wittenberg, E. Widiasih, S. Zhao, Revisiting ENSO/Indian Ocean Dipole phase relationships. *Geophys. Res. Lett.* **44**, 2481–2492 (2017).

22. F. Jiang, W. Zhang, F. F. Jin, M. F. Stuecker, A. Timmermann, M. J. McPhaden, J. Boucharel, A. T. Wittenberg, Resolving the Tropical Pacific/Atlantic interaction conundrum. *Geophys. Res. Lett.* **50**, e2023GL103777 (2023).

23. B. Qin, Z. Yang, M. Mu, Y. Wei, Y. Cui, X. Fang, G. Dai, S. Yuan, The first kind of predictability problem of El Niño predictions in a multivariate coupled data-driven model. *Q. J. Roy. Meteorol. Soc.* **150**, 5452–5471 (2024).

24. V. Eyring, S. Bony, G. A. Meehl, C. A. Senior, B. Stevens, R. J. Stouffer, K. E. Taylor, Overview of the Coupled Model Intercomparison Project Phase 6 (CMIP6) experimental design and organization. *Geosci. Model Dev.* **9**, 1937–1958 (2016).

25. M. Ravichandran, D. Behringer, S. Sivareddy, M. S. Girishkumar, N. Chacko, R. Harikumar, Evaluation of the global ocean data assimilation system at INCOIS: The tropical Indian Ocean. *Ocean Model.* **69**, 123–135 (2013).

26. D. Behringer, M. Ji, A. Leetmaa, An improved coupled model for ENSO prediction and implications for ocean initialization. Part I: The ocean data assimilation system. *Mon. Weather Rev.* **126**, 1013–1021 (1998).

27. B. P. Kirtman, D. Min, J. M. Infanti, J. L. Kinter, D. A. Paolino, Q. Zhang, H. van den Dool, S. Saha, M. P. Mendez, E. Becker, P. T. Peng, P. Tripp, J. Huang, D. G. DeWitt, M. K. Tippett, A. G. Barnston, S. H. Li, A. Rosati, S. D. Schubert, M. Rienecker, M. Suarez, Z. E. Li, J. Marshak, Y. K. Lim, J. Tribbia, K. Pegion, W. J. Merryfield, B. Denis, E. F.

Wood, The North American multimodel ensemble: Phase-1 seasonal-to-interannual prediction; phase-2 toward developing intraseasonal prediction. *Bull. Am. Meteorol. Soc.* **95**, 585–601 (2014).

28. H. Bellenger, E. Guilyardi, J. Leloup, M. Lengaigne, J. Vialard, ENSO representation in climate models: From CMIP3 to CMIP5. *Clim. Dyn.* **42**, 1999–2018 (2013).

29. G. Li, S. P. Xie, Y. Du, Y. Y. Luo, Effects of excessive equatorial cold tongue bias on the projections of tropical Pacific climate change. Part I: The warming pattern in CMIP5 multi-model ensemble. *Clim. Dyn.* **47**, 3817–3831 (2016).

30. T. Iwakiri, M. Watanabe, Mechanisms linking multi-year La Niña with preceding strong El Niño. *Sci. Rep.* **11**, 17465 (2021).

31. X. F. Li, Z. Z. Hu, Y. H. Tseng, Y. Y. Liu, P. Liang, A historical perspective of the La Niña event in 2020/2021. *J. Geophys. Res. Atmos.* **127**, e2021JD035546 (2022).

32. C. Model, F.-F. Jin, An equatorial ocean recharge paradigm for ENSO. Part I: Conceptual model. *J. Atmos. Sci.* **54**, 811–829 (1997).

33. F.-F. Jin, An equatorial ocean recharge paradigm for ENSO. Part II: A stripped-down coupled model. *J. Atmos. Sci.* **54**, 830–847 (1997).

34. C. Gao, M. Chen, L. Zhou, L. Feng, R.-H. Zhang, The 2020–2021 prolonged La Niña evolution in the tropical Pacific. *Sci. China Earth Sci.* **65**, 2248–2266 (2022).

35. K. Bi, L. Xie, H. Zhang, X. Chen, X. Gu, Q. Tian, Accurate medium-range global weather forecasting with 3D neural networks. *Nature* **619**, 533–538 (2023).

36. Y. Zhang, M. Long, K. Chen, L. Xing, R. Jin, M. I. Jordan, J. Wang, Skilful nowcasting of extreme precipitation with NowcastNet. *Nature* **619**, 526–532 (2023).

37. L. Zhou, R.-H. Zhang, L. Tao, AI-Enabled conditional nonlinear optimal perturbation enhances ensemble prediction of extreme El Niño events. *npj Clim. Atmos. Sci.* **9**, 30 (2026).

38. C. Bodnar, W. P. Bruinsma, A. Lucic, M. Stanley, A. Allen, J. Brandstetter, P. Garvan, M. Riechert, J. A. Weyn, H. Dong, J. K. Gupta, K. Thambiratnam, A. T. Archibald, C.-C. Wu, E. Heider, M. Welling, R. E. Turner, P. Perdikaris, A foundation model for the Earth system. *Nature* **641**, 1180–1187 (2025).

39. A. Vaswani, N. Shazeer, N. Parmar, J. Uszkoreit, L. Jones, A. N. Gomez, L. Kaiser, I. Polosukhin, Attention is all you need. *Adv. Neural Inf. Proces. Syst.* **30**, 6000–6010 (2017).

40. A. Dosovitskiy, L. Beyer, A. Kolesnikov, D. Weissenborn, X. Zhai, T. Unterthiner, M. Dehghani, M. Minderer, G. Heigold, S. Gelly, An image is worth 16×16 words: Transformers for image recognition at scale. arXiv:2010.11929, [cs.CV] (2020).

41. G. Bertasius, H. Wang, L. Torresani, Is space-time attention all you need for video understanding? in *International Conference on Machine Learning (ICML)*, (2021).

42. R.-H. Zhang, L. Zhou, C. Gao, L. Tao, A transformer-based coupled ocean-atmosphere model for ENSO studies. *Sci. Bull.* **15**, 2323–2327 (2024).

43. L. Zhou, R.-H. Zhang, The 3D-Geoformer for ENSO studies: A transformer-based model with integrated gradient methods for enhanced explainability. *J. Oceanol. Limnol.* **43**, 1688–1708 (2025).

44. M. Sundararajan, A. Taly, Q. Yan, "Axiomatic attribution for deep networks" in *ICML'17: Proceedings of the 34th International Conference on Machine Learning* (2017), vol. 70, pp. 3319–3328.

45. N. A. Hasan, Y. Chikamoto, M. J. McPhaden, The influence of tropical basin interactions on the 2020-2022 double-dip La Niña. *Front. Clim.* **4**, 1001174 (2022).

46. J. C. H. Chiang, A. H. Sobel, Tropical tropospheric temperature variations caused by ENSO and their influence on the remote tropical climate. *J. Climate* **15**, 2616–2631 (2002).

47. S. Liu, P. Chang, X. Wan, S. G. Yeager, I. Richter, Role of the maritime continent in the remote influence of Atlantic Niño on the Pacific. *Nat. Commun.* **14**, 3327 (2023).

48. L. Fan, X. Meng, The asymmetric predictive power of indian ocean dipole for subsequent Year's ENSO: Role of Atlantic Ocean as an intermediary. *Geophys. Res. Lett.* **50**, e2023GL105525 (2023).
